# Long noncoding RNA ATB promotes the epithelial−mesenchymal transition by upregulating the miR-200c/Twist1 axe and predicts poor prognosis in breast cancer

**DOI:** 10.1038/s41419-018-1210-9

**Published:** 2018-12-05

**Authors:** Rong-Hui Li, Min Chen, Jing Liu, Chang-Chun Shao, Cui-Ping Guo, Xiao-Long Wei, Yao-Chen Li, Wen-He Huang, Guo-Jun Zhang

**Affiliations:** 1 ChangJiang Scholar’s Laboratory, Shantou University Medical College (SUMC), 515041 Shantou, Guangdong China; 2 The Cancer Center, Xiang’an Hospital of Xiamen University, 2000 East Xiang’an Rd., Xiang’an, Xiamen China; 3 The Breast Center, The Cancer Hospital of SUMC, 515041 Shantou, Guangdong China; 4 Department of Pathology, The Cancer Hospital of SUMC, Shantou, Guangdong China

## Abstract

Recent studies indicate that the long noncoding RNA ATB (lncATB) can induce the epithelial−mesenchymal transition (EMT) in cancer cells, but the specific cellular targets of lncATB require further investigation. In the present study, the upregulation of lncATB in breast cancer cells was validated in a TGF-β-induced EMT model. Gain- and loss-of-function studies demonstrated that lncATB enhanced cell migration, invasion and clonogenicity in vitro and in vivo. LncATB promoted the EMT by acting as a sponge for the miR-200 family and restoring Twist1 expression. Subsequently, the clinical significance of lncATB was investigated in a cohort of breast cancer patients (*N* = 131). Higher lncATB expression was correlated with increased nodal metastasis (*P* = 0.036) and advanced clinical stage (*P* = 0.011) as well as shorter disease-free survival (*P* = 0.043) and overall survival (*P* = 0.046). These findings define Twist1 as a major target of lncATB in the induction of the EMT and highlight lncATB as a biomarker in breast cancer patients.

## Introduction

Over the past decade, great improvements have been made in the management of breast cancer, but patient outcomes still merit consideration based on the increasing rate of tumour incidence and the high rate of tumour-specific death due to recurrence and metastasis^1–3^. However, targeted therapy has proven to be an effective strategy for breast cancer treatment^4,5^. Therefore, identifying new targets that contribute to breast cancer malignancy, as well as elucidating the underlying molecular mechanisms, is urgently required.

At least 75% of the genome is transcribed into noncoding RNA, which has no protein-coding capacity^6^. Generally, long noncoding RNAs (lncRNAs) are longer than 200 nucleotides^7–9^ and undergo transcription, 5′-capping, polyadenylation and splicing under the control of RNA polymerase II^6^. LncRNAs participate in the regulation of gene expression at the transcriptional and post-transcriptional levels^10–12^. LncRNAs drive various cancerous phenotypes, including characteristics associated with immortality, cancer cell proliferation, anti-apoptosis and invasion, through the interaction of these RNAs with other cellular macromolecules, such as DNA, protein, and RNA^13–15^.

To our knowledge, several lncRNAs associated with breast cancer prognosis have been identified, including HOTAIR, which regulates cancer cell proliferation and invasion^16,17^, MALAT1, which has been correlated with breast cancer metastasis^18,19^, H19, which is involved in various functions during the complex process of tumour progression^20–22^, and other CCAT2^23,24^, MCM3AP-AS1 and PCAT^25^. Very recently, using Gene Expression Omnibus (GEO) and the Cancer Genome Atlas (TCGA) database, Xu et al.^25^ group identified TINCR, LINC00511, and PPP1R26-AS1 as subtype-specific lncRNAs associated with HER-2, triple-negative and luminal B subtypes, respectively. Intriguingly, an increasing number of lncRNAs have been validated as potential breast cancer biomarkers, including the four potentially diagnostic lncRNAs RP11-434D9.1, LINC00052, BC016831, and IGKV^26^, and BCAR4 as a prognostic marker for tamoxifen resistance and tumour invasion^24,27,28^. Thus, all these findings highlight lncRNAs as potential biomarkers for breast cancer as well as the mechanisms contributing to breast cancer malignancy^22,29–31^.

LncATB, a noncoding RNA located on chromosome 14 (ENST00000493038) with a length of 2446-bp, was first reported as a noncoding transcript enriched in human hepatocellular carcinoma (HCC) cells after a TGF-β stimulus in 2014, and also as a predictor for HCC patient survival^32^. Since its discovery in HCC, lncATB has been linked as a novel potential diagnostic and prognostic biomarker to other human tumours, such as non-small cell lung cancer^33^, osteosarcoma^34^, colon cancer^35^, renal cell carcinoma^36,37^, gastric cancer^38,39^, thyroid cancer^40^, oesophageal squamous cell carcinoma^41^ and glioma malignancy^42^. In an effort to explore the role of lncATB, we found when searching the NONCODE database (http://www.noncode.org) that the expression of lncATB was higher in the breast and ovary. Additional evidence has accumulated revealing the premetastatic role of lncATB in human cancer via acting as a sponge for the miR-200 family to thereby restore ZEB1and ZEB2 expression^32,34,43,44^, restore ZNF217 expression^43^ or promote IL-11 signalling^32^. Regarding the role of lncATB in breast cancer, a recent study reported that lncATB promoted trastuzumab resistance and invasiveness in Her2-positive breast cancer cells by competitively binding miR-200c to restore the expression of the miR-200c target genes *ZEB1* and *ZNF-217*, resulting in EMT-mediated drug resistance^45^. However, the roles and clinical significance of lncATB, the functions of lncATB in malignancy and the related molecular mechanisms in breast remain unclear.

In the present study, we aimed to explore the role of lncATB in the EMT and the relevant mechanisms of lncATB in breast cancer progression and metastases, and to further identify the clinical significance of lncATB in a breast cancer cohort.

## Results

### TGF-β induced lncATB and altered EMT markers in breast cancer cells in vitro

**Fig. 1:**
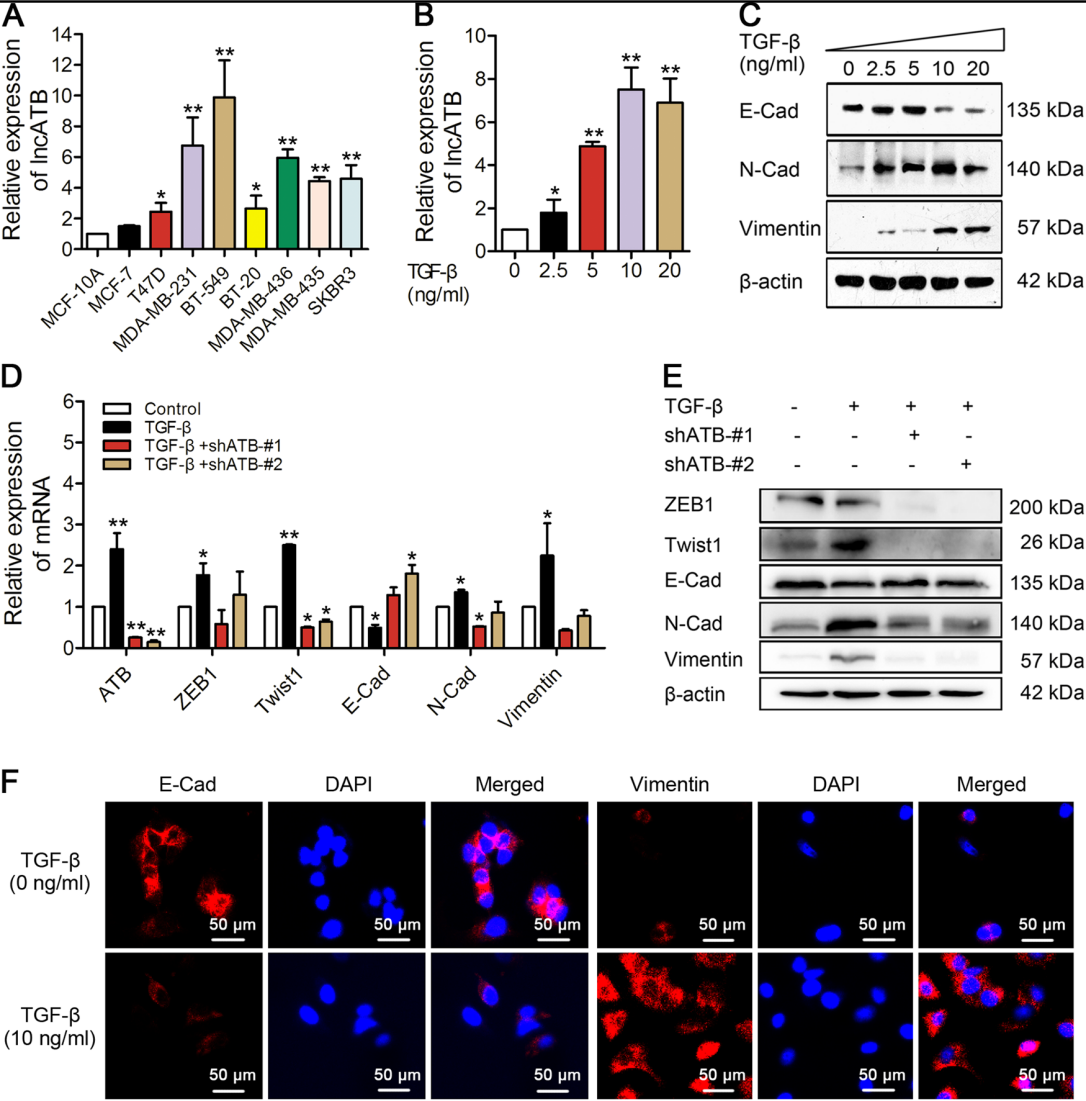
TGF-β induced lncATB and altered EMT markers in MCF-7 cells. **a** LncATB expression in different breast cancer cell lines. **b** Relative expression of lncATB in MCF-7 cells treated with TGF-β at the indicated concentrations. **c** The protein levels of EMT markers determined by western blot analysis in MCF-7 cells treated with TGF-β at the indicated concentrations. **d** Relative mRNA levels of lncATB and EMT markers measured by qRT-PCR assays in MCF-7 cells treated with TGF-β or both treated with TGF-β and shATB-#1/2. **e** Relative protein levels of EMT markers measured by western blot analysis in MCF-7 cells treated with TGF-β or both treated with TGF-β and shATB-#1/2. **f** Immunofluorescence staining of E-Cad and Vimentin in MCF-7 cells treated with 10 ng/µl TGF-β. *P < 0.05 and **P < 0.01

To detect lncATB levels, qRT-PCR assays were used to determine the relative expression of lncATB in the normal human breast cell line MCF-10A and eight breast cancer cell lines. Compared to the normal breast cell line MCF-10A, the expression level of lncATB was higher in breast cancer cell lines. Even though the variation for the lncATB expression level was noticed in different breast cancer cell lines, however, the average expression level of lncATB was relative higher in four TNBC cell lines (MDA-MB-231, MDA-MB-436, BT-549 and BT-20) and two Her-2-positive cell lines (SKBR3 and MDA-MB-435) than two luminal A cell lines (MCF-7 and T47D) (Fig. 1a).

To determine whether lncATB can be regulated by TGF-β, we continuously treated MCF-7 cells with TGF-β at various concentrations (0, 2.5, 5, 10, or 20 ng/ml) for 72 h. The expression of lncATB was sufficiently upregulated with TGF-β treatment in a dose-dependent manner (Fig. 1b) and treatment with 10 ng/ml TGF-β resulted in a saturation level. And also the downregulation of E-Cadherin (E-Cad) protein or upregulation of N-cadherin (N-Cad) and Vimentin proteins was shown to be dose-dependent regarding TGF-β treatment (Fig. 1c). In addition, the 10 ng/ml TGF-β treatment decreased the expression levels of an epithelial marker E-Cad, but increased the expression of the mesenchymal markers N-Cad and Vimentin and the transcription factors ZEB1 and Twist1, and importantly, through knockdown lncATB rescued the TGF-β-induced dysregulation of those genes (Fig. 1d, e). Moreover, immunofluorescence staining revealed that 10 ng/ml TGF-β reduced E-Cad expression and induced Vimentin expression in MCF-7 cells (Fig. 1e). These results demonstrated that TGF-β, an EMT inducer, induced lncATB and altered EMT markers in breast cancer cells in vitro.

### LncATB overexpression and knockdown promoted and suppressed EMT, respectively, in breast cancer cells

**Fig. 2:**
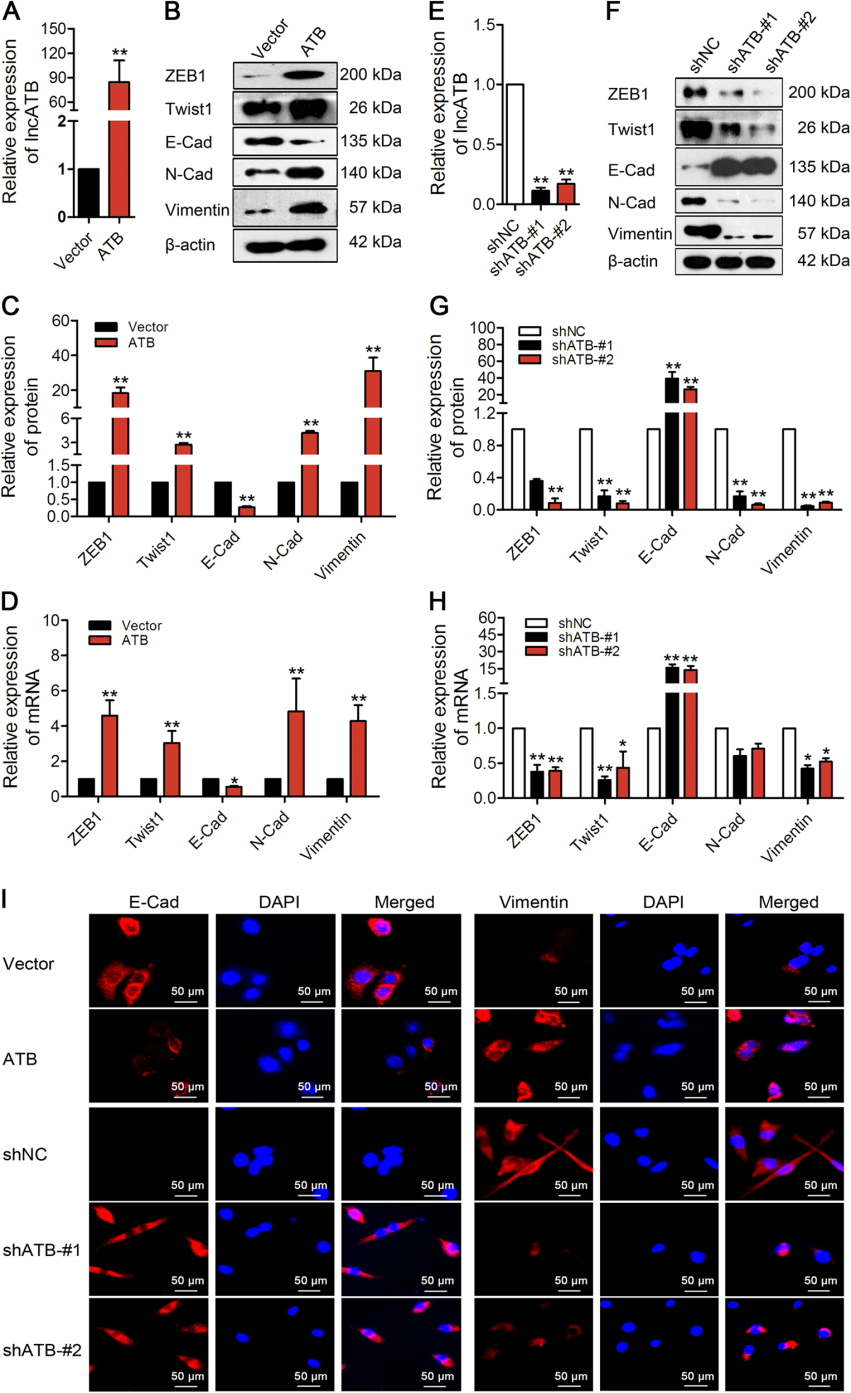
LncATB overexpression increased mesenchymal makers and lncATB knockdown increased epithelial markers in breast cancer cells. **a** LncATB overexpression in stable MCF-7 cell clones. **b**, **c** The protein levels of EMT markers determined by western blot analysis in lncATB-overexpressing MCF-7 cells. **d** The mRNA levels of EMT markers in lncATB-overexpressing MCF-7 cells. **e** Knocking down lncATB in stable BT-549 cell clones. **f**, **g** The protein levels of EMT markers determined by western blot analysis in lncATB-knockdown BT-549 cells. **h** The mRNA levels of EMT markers in lncATB-knockdown BT-549 cells. **i** Immunofluorescence staining of E-Cad and Vimentin in MCF-7-ATB and BT-549-shATB cells. *P < 0.05 and **P < 0.01

To investigate the potential role of lncATB in the EMT process in breast cancer cells, we overexpressed (Supplementary Fig. S1a) or knocked down lncATB in breast cancer cell lines. Successful ectopic lncATB expression (84.4-fold increase in MCF-7-ATB stable cells, Fig. 2a) altered the expression of both epithelial and mesenchymal markers at both the protein (Fig. 2b, c) and transcriptional levels (Fig. 2d), e.g., upregulated ZEB1, Twist1, N-Cad and Vimentin and downregulated E-Cad were observed. Successful lncATB knockdown (88.7 and 82.9% knockdown for lncATB using shATB-#1 and shATB-#2, respectively, in BT-549 cells, Fig. 2e) resulted in downregulated ZEB1, Twist1 and Vimentin and upregulated E-Cad at both the protein (Fig. 2f, g) and transcriptional levels (Fig. 2h). Furthermore, as expected, immunofluorescence staining showed that E-Cad was decreased and Vimentin was increased in the MCF-7-ATB stable cells, whereas E-Cad was increased and Vimentin was decreased in the BT-549-shATB stable cells (Fig. 2i). Additionally, other EMT-related markers, such as MMP-2, MMP-9, Snail, and Slug, were assessed but did not have a significant relationship with lncATB (Supplementary Fig. S1b, c). These data indicated that the transcription factors ZEB1 and Twist1 were upregulated by lncATB and promoted the EMT in breast cancer cells.

### LncATB acts as a miR-200 family sponge

**Fig. 3:**
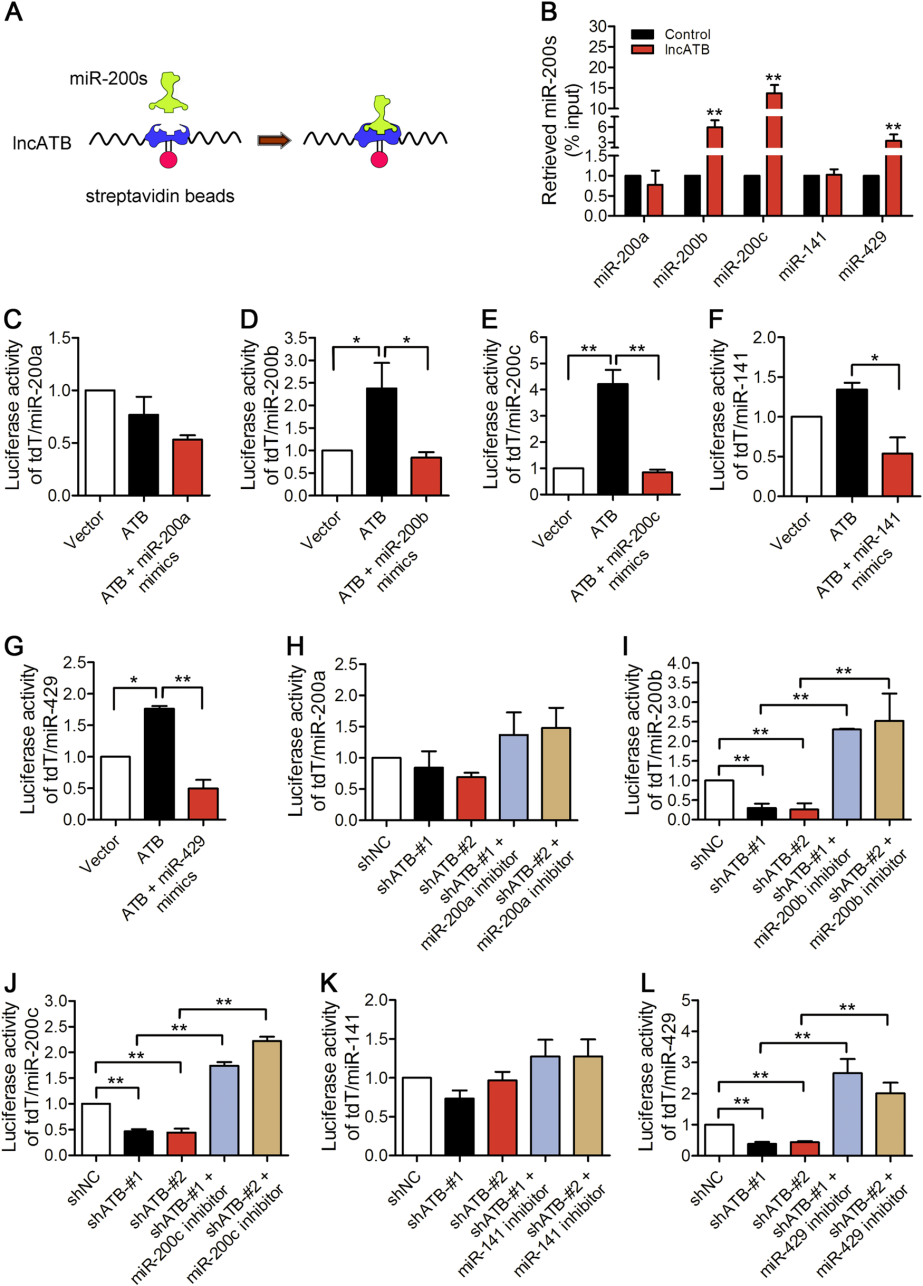
LncATB functions by binding to miR-200 family members. **a** RNA pull-down followed by microRNA qRT-PCR assays to detect miR-200 family members endogenously associated with lncATB. **b** MCF-7 cell lysates were incubated with biotin-labelled lncATB; after the pull-down, miR-200 miRNAs were extracted and assessed by qRT-PCR assays. **c**−**g** Relative luciferase activity in MCF-7 cells transfected with specific miR-200 luciferase reporters in the presence of pcDNA3.1-ATB alone or with the cotransfection of **c** miR-200a mimics, **d** miR-200b mimics, **e** miR-200c mimics, **f** miR-141 mimics, or **g** miR-429 mimics. **h**−**l** Relative luciferase activity in BT-549 cells cotransfected with specific miR-200 luciferase reporters and shATB-#1/2. For each specific reporter, **h** miR-200a inhibitor, **i** miR-200b inhibitor, **j** miR-200c inhibitor, **k** miR-141 inhibitor, or **l** miR-429 inhibitor was also added. *P < 0.05 and **P < 0.01

We used the NCBI and miRBase programs to predict the miRNAs that potentially bind to lncATB. The analysis showed that lncATB has three potential binding sites for the miR-200 family. To further prove the direct binding between lncATB and the miR-200 family, we applied biotin-labelled lncATB RNA probes to pull down endogenous miR-200 family members in MCF-7 cells (Fig. 3a). The RNA pull-down experiment showed that the strongest association between members of the miR-200 family and lncATB was with miR-200c (13.8-fold), followed by miR-200b and miR-429 (Fig. 3b). Surprisingly, the expression levels of the miR-200 family members did not significantly change upon lncATB overexpression or deletion, indicating that lncATB may only be a sponge for miR-200 family (Supplementary Fig. S1d, e). Additionally, a luciferase reporter system, tdT/miR-200 family, was also used to confirm the competitive binding between lncATB and the miR-200 family. Firstly, we verified the activity of the reporter genes, which exhibited signalling in a dose-dependent manner (Supplementary Fig. S2a-j). The results showed that the luciferase activity of tdT/miR-200b, tdT/miR-200c and tdT/miR-429 increased after lncATB overexpression in MCF-7 cells and decreased after lncATB deletion in BT-549 cells in a dose-dependent manner, while there was no significant difference in the luciferase activity of tdT/miR-200a or tdT/miR-141 (Supplementary Fig. S2k-t). The ectopic expression of miR-200b, miR-200c or miR-429 abrogated this increase (Fig. 3c–g), and conversely, the inhibition of miR-200b, miR-200c or miR-429 overcame this decrease (Fig. 3h–l). All of these results demonstrated that lncATB acts as a miR200-family sponge in breast cancer cells, especially for miR-200c.

### LncATB restores the expression of Twist1 by binding to miR-200c

**Fig. 4:**
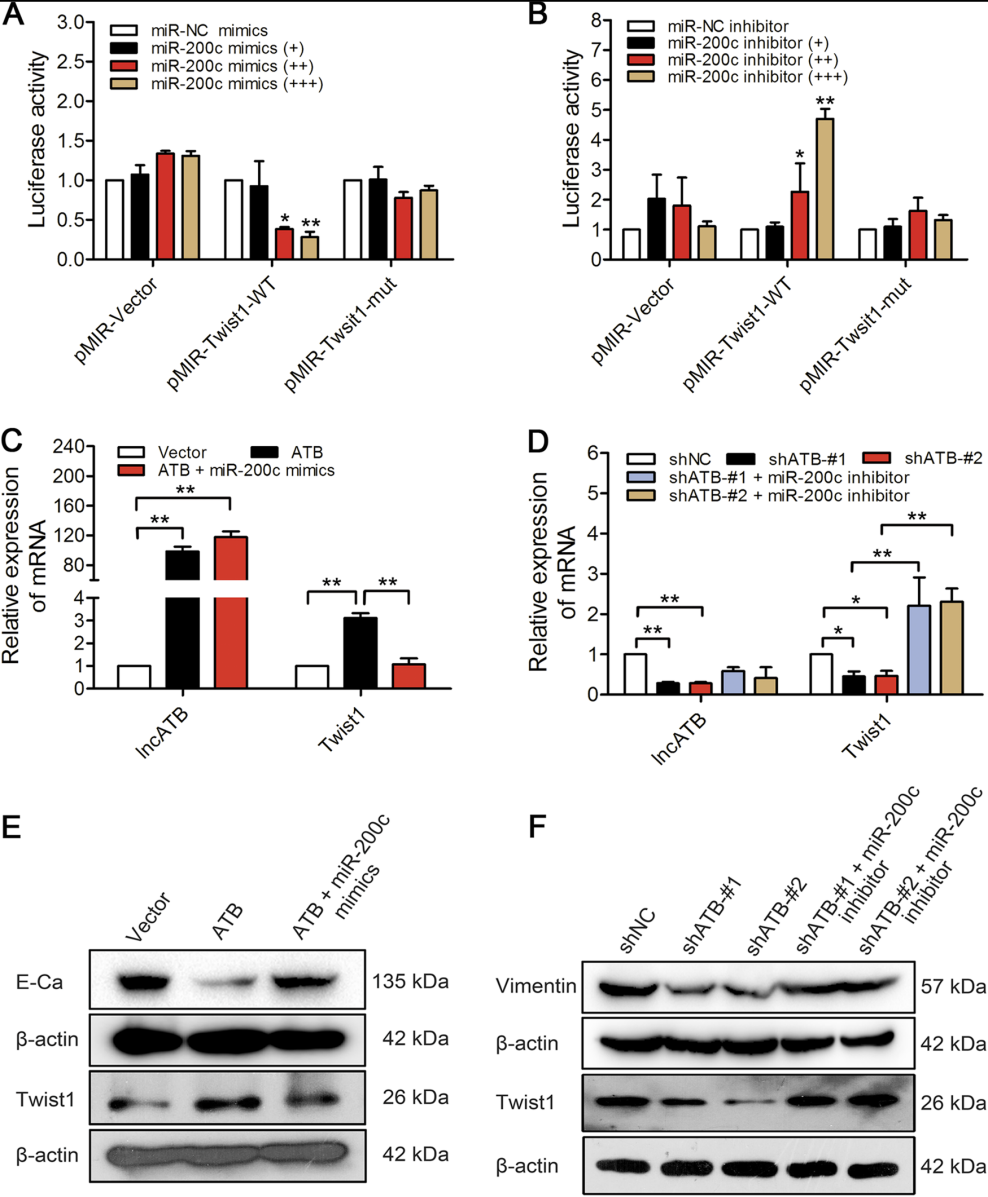
Regulation of Twist1 mediated by lncATB via binding to miR-200c. **a** Luciferase activity in MCF-7 cells cotransfected with luciferase reporter (pMIR-Vector, pMIR-Twist1-WT, or pMIR-Twist1-mut) and miR-200c mimics with a dose dependent. **b** Luciferase activity in BT-549 cells cotransfected with luciferase reporter (pMIR-Vector, pMIR-Twist1-WT, or pMIR-Twist1-mut) and miR-200c inhibitor with a dose dependent. **c** The mRNA levels of Twist1 in MCF-7 cells overexpressing lncATB or ectopically expressing both lncATB and miR-200c. **d** The mRNA levels of Twist1 in BT-549 cells with downregulated lncATB or with both shATB and miR-200c inhibitor. **e** The protein levels of Twist1 and E-Cad in MCF-7 cells overexpressing lncATB or ectopically expressing both lncATB and miR-200c. **f** The protein levels of Twist1 and Vimentin in BT-549 cells with downregulated lncATB or with both shATB and miR-200c inhibitor. *P < 0.05 and **P < 0.01

When searching the microRNA databases miRBase, TargetScanHuman and the miRcode program, we confirmed that there is one binding site for miR-200c in the Twist1 3′UTR (Supplementary Table S1). Thus, we constructed two luciferase reporter vectors containing the wild-type or mutant Twist1 3′UTR. When miR-200c mimics was administered, the luciferase activity of WT reporter was decreased in a dose-dependent manner (Fig. 4a). In contrast to miR-200c mimics, the luciferase activity of WT reporter was conversely increased by adding miR-200c inhibitor (Fig. 4b). However, the luciferase activity of mutant reporter did not change to the miR-200c mimics or inhibitors. The results showed that lncATB-induced Twist1 mRNA (Fig. 4c) or protein and lncATB-suppressed E-Cad protein (Fig. 4e) expression in MCF-7 cells was abrogated by the ectopic expression of miR-200c. Conversely, in BT-549 cells with knocked-down lncATB, the decreased Twist1 mRNA (Fig. 4d) or protein and Vimentin protein (Fig. 4f) expression was rescued by inhibiting miR-200c. All of these data revealed the important role of lncATB in restoring Twist1 expression by acting as a sponge for miR-200c.

### LncATB promotes breast cancer cell proliferation, migration and invasion

**Fig. 5:**
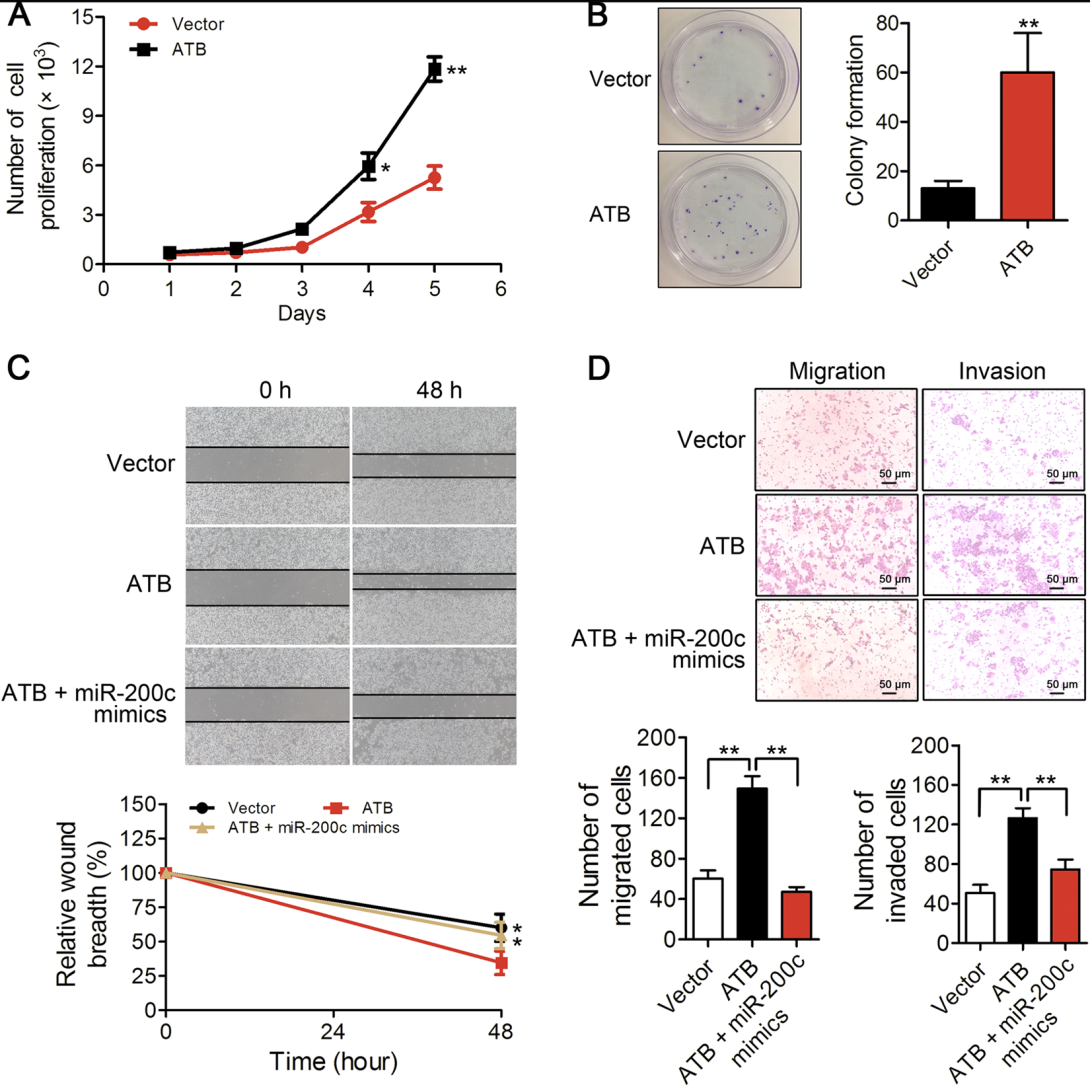
LncATB promotes cancer progression in MCF-7 cells. **a** Cell proliferation of stably lncATB-overexpressing and control MCF-7 cells as assessed by the CCK-8 assay. **b** Colony numbers were counted in the MCF-7-ATB and control MCF-7 cell groups. **c** Wound-healing assay to evaluate the effect of lncATB on cell migration in groups of control MCF-7, MCF-7-ATB, or cells overexpressing both lncATB and miR-200c. **d** The migrated (left) and invaded (right) cell numbers for the groups of control MCF-7, MCF-7-ATB cells, or cells overexpressing both lncATB and miR-200c were counted 48 h after seeding. Scale bar = 50 μm. *<0.05 and **<0.01

To directly test whether lncATB promotes breast cancer progression, we conducted proliferation, colony formation, wound-healing, and migration and invasion assays in the MCF-7-ATB and BT-549-shATB cells. We observed that the forced overexpression of lncATB expedited proliferation by 3-fold (Fig. 5a) and increased the number of colonies by 4.6-fold **(**Fig. 5b). Furthermore, tumour cell migration and invasion were increased approximately twofold in the MCF-7-ATB cells, while the increase was abrogated when the cells were treated with miR-200c mimics (Fig. 5c, d).

**Fig. 6:**
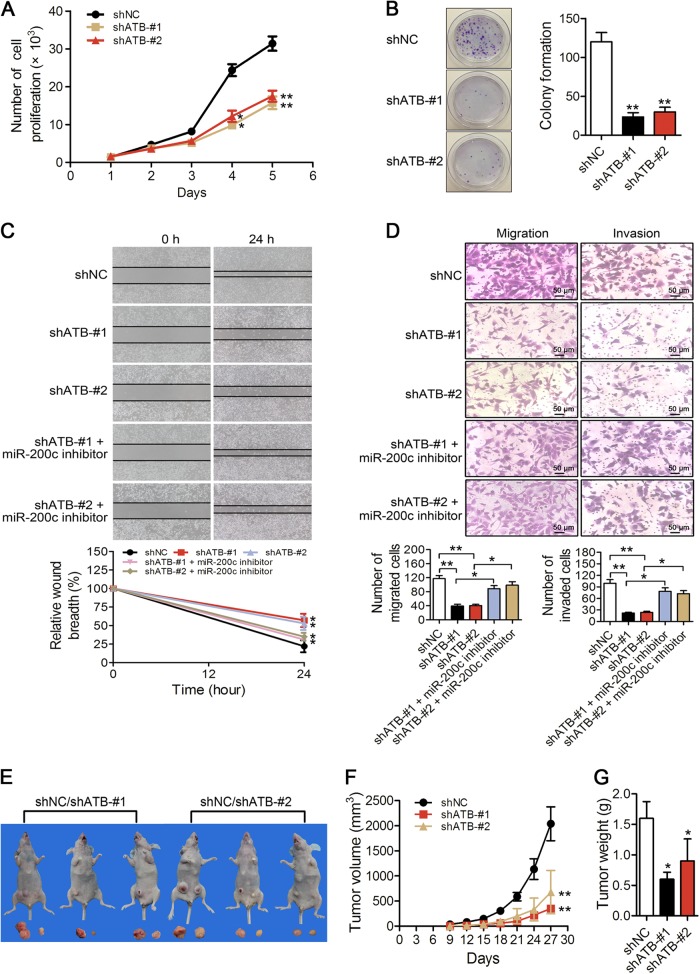
LncATB deletion reduced breast cancer progression in vitro and in vivo. **a** Cell viability assay in BT-549 cells with knocked down lncATB. **b** Colony numbers were counted in the BT-549-shATB and control BT-549-shNC cell groups. **c** Wound-healing assay to evaluate the effect of lncATB on cell migration in the BT-549-shATB and control BT-549-shNC cell groups. **d** The migrated (left) and invaded (right) cell numbers in the groups of control BT-549-shNC cells, BT-549-shATB cells, and cells with both lncATB and miR-200c inhibition were counted 24 h after seeding. **e** For each mouse, shNC cells and lncATB-depleted cells were subcutaneously implanted into the left and right fourth mammary pad, respectively. There were two groups: shNC/shATB-#1 and shNC/shATB-#2, each group has three mice. **f** Tumours were measured every 3 days with external callipers, and the tumour volume was calculated according to the following formula: Volume = 0.5 × *a*^2^ × b, where ‘*a*’ is the smallest superficial diameter and ‘*b*’ is the largest superficial diameter. **g** Twenty-seven days after the tumour was implanted, the tumours were excised and weighed. Scale bar = 50 μm. *P < 0.05 and **P < 0.01

The depletion of lncATB suppressed cell growth by approximately 2-fold (Fig. 6a) and decreased the number of colonies by 4−5-fold (Fig. 6b). Additionally, the depletion of lncATB reduced the migration and invasion ability by 2.5-fold in BT-549 cells, while this decrease was rescued when the cells were treated with miR-200c inhibitor (Fig. 6c, d). All of these results implied that lncATB has the potential to promote breast cancer progression and metastasis.

### LncATB induces tumourigenesis in breast cancer tumour xenograft models in vivo

To ascertain the effects of lncATB expression in tumourigenesis, we performed tumour xenograft studies with highly tumourigenic MDA-MB-231 cells in nude mice. Firstly, we detected the relative expression levels of lncATB and EMT markers. The results led to a conclusion similar to that above; i.e., when lncATB was depleted, the mRNA expression levels of ZEB1, Twist1 and Vimentin were increased while that of E-Cad was decreased (Supplementary Fig. S3a, b). Next, control or stably lncATB-depleted MDA-MB-231 cells were subcutaneously implanted into the left or right fourth breast of nude mice (Fig. 6e). Tumour growth was determined using callipers, and the tumour volume was calculated as *V* = 0.5 × width^2^ × length. At 27 days after implantation, an 82 and 73% reduction in the tumour volume was observed in the lncATB-downregulated shATB-#1 and shATB-#2 groups, respectively, compared with the control (Fig. 6f). At the end of the observation period, the subcutaneous tumours were dissected and weighed. Our results showed that lncATB downregulation reduced tumour weights than those in the control, with a 62% reduction observed in the shATB-#1 group and a 46% reduction observed in the shATB-#2 group (Fig. 6g). The immunohistochemistry results revealed that E-Cad expression was increased and Vimentin expression was decreased (Supplementary Fig. S3c). We extracted total RNA from the mouse tumour tissues, and the qRT-PCR assay results suggested that the ZEB1 and Twist1 expression levels were reduced (Supplementary Fig. S3d, e). These results indicated that lncATB downregulation inhibited tumour growth in vivo.

### LncATB is upregulated in breast cancer tissues and is associated with a poor prognosis in breast cancer patients

**Fig. 7:**
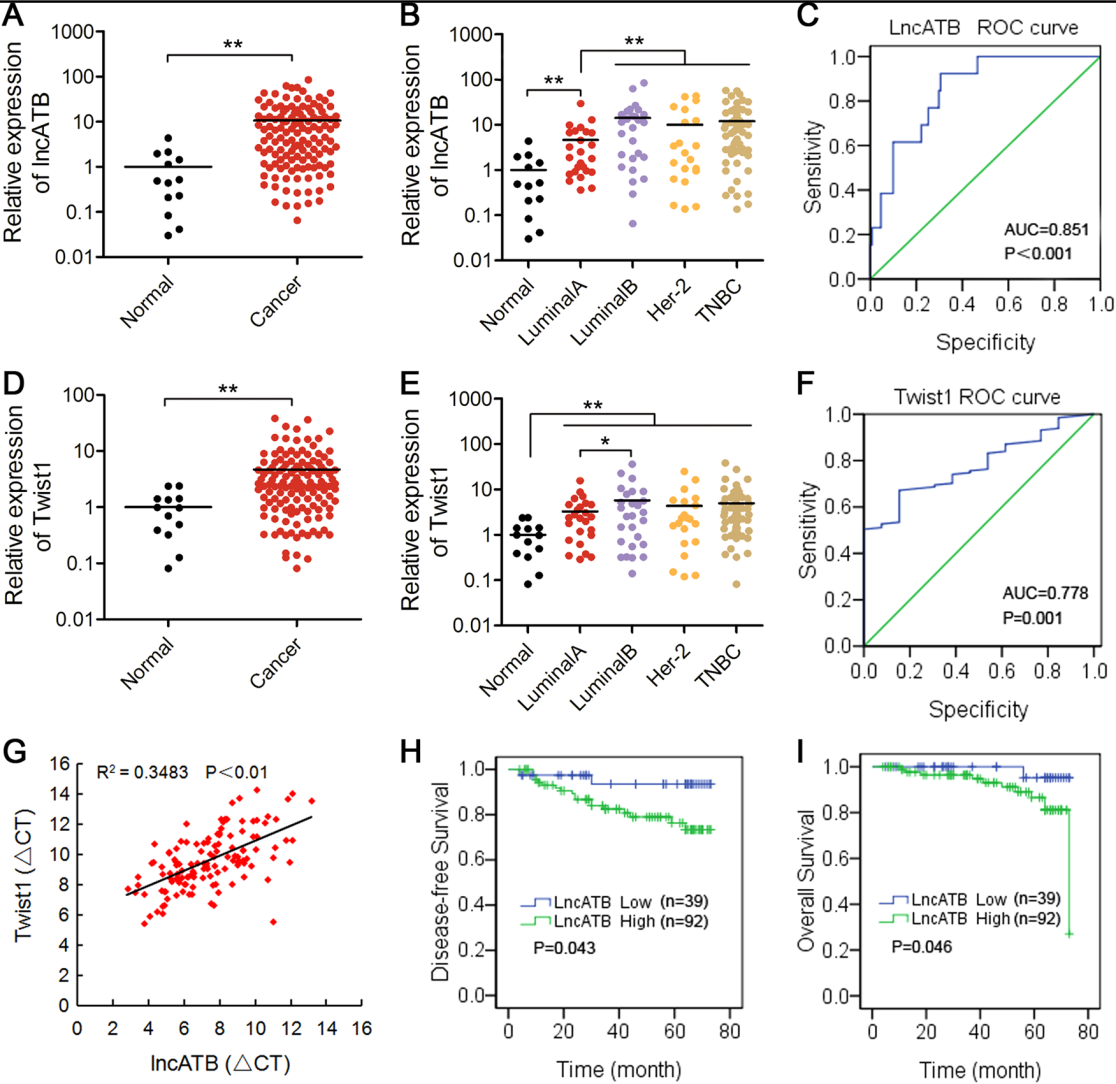
High levels of lncATB indicated poor prognosis in breast cancer patients. **a** The relative expression of lncATB in normal breast and breast cancer tissues. **b** The relative expression of lncATB in different breast cancer tissue subtypes. **c** Assessment of the diagnostic efficacy of lncATB in breast cancer patients by calculating the area under the ROC curve (AUC = 0.851, *P* < 0.001). **d** The relative expression of Twist1 in normal breast and breast cancer tissues. **e** The relative expression of Twist1 in different breast cancer tissue subtypes. **f** Assessment of the diagnostic efficacy of Twist1 in breast cancer patients by calculating the area under the ROC curve (AUC = 0.778, *P* = 0.001). **g** The correlation between the lncATB transcript level and Twist1 mRNA levels was measured in 131 breast cancer tissues. **h** Disease-free survival for the two groups defined by low and high expression of lncATB in breast cancer patients. **i** Overall survival for the two groups defined by low and high expression of lncATB in breast cancer patients. *P < 0.05 and **P < 0.01

Firstly, we searched the lncRNA database NONCODE and found that normal lncATB expression was higher in breast and ovarian tissues than in other organs (Supplementary Fig. S4a, b). To further define the role of lncATB in human breast cancer patients and the correlation between lncATB and Twist1, we measured the relative expression levels of lncATB and Twist1 in 146 samples, including 15 normal breast tissues and 131 breast cancer tissues. Clinical data on the lncATB (Fig. 7a, b) and Twist1 (Fig. 7d, e) expression level revealed that higher expression level observed in triple-negative and Luminal B (Her-2 positive) breast cancer, relative lower level in Luminal A breast cancer, and the lowest level in normal mammary glands. The diagnostic efficacy of lncATB (AUC = 0.851, *P* < 0.001, Fig. 7c) and Twist1 (AUC = 0.778, *P* = 0.001, Fig. 7f) in breast cancer patients was evaluated by calculating the area under the receiver operating characteristic (ROC) curve. Moreover, we also found that the lncATB transcript level was significantly correlated with the Twist1 (Fig. 7g) mRNA levels. Patients were classified based on the level of lncATB expression in tissues into the low expression group (*n* = 39) and the high expression group (*n* = 92). Then, we analysed the disease-free survival (DFS) and the overall survival (OS) using a Kaplan−Meier analysis between the two groups. The results showed that the DFS (*χ*^2^ = 4.099, *P* = 0.043) and OS (*χ*^2^ = 3.988, *P* = 0.046) were longer in the low-lncATB-expression group than in the high-lncATB-expression group (Fig. 7h, i). However, there was no significant difference in the DFS or OS between the groups with high and low expression levels of Twist1 (Supplementary Fig. S5a, b). Similarly, we further evaluated the prognostic value of low or high expression of Twist1 mRNA in Kaplan−Meier Plotter database of breast cancer. The expression of Twist1 was not significantly related to RFS (*P* = 0.7, Supplementary Fig. S5c) and OS (*P* = 0.31, Supplementary Fig. S5d). In our study, we have further analysed the survival of both lncATB and Twist1 in breast cancer patients. There was no statistically significant difference between DFS (*P* = 0.174, Supplementary Fig. S5e) and OS (*P* = 0.170, Supplementary Fig. S5f), but the results tend to that breast cancer patients have a shorter survival when both lncATB and Twist1 were both positively expressed. Thus, these results indicate that lncATB might be a new poor prognostic marker for breast cancer with potential oncogenic characteristics.

### The clinicopathological features associated with low and high lncATB and Twist1 expression in breast cancer patients

We further assessed the correlation between the lncATB expression level and the clinicopathological characteristics of 131 breast cancer samples, as summarized in Table 1. The percentage of patients with histological grade III breast cancer was 67.4% in the high-lncATB-expression group compared with 43.6% in the low-lncATB-expression group (*χ*^2^ = 6.482; *P* = 0.011). Additionally, the rate of Ki67 positivity was 92.4% in the high-lncATB-expression group and 56.4% in the low-lncATB-expression group (*χ*^2^ = 23.696; *P* < 0.001). In total, 18 out of 92 patients (19.6%) with high lncATB expression exhibited a significantly higher rate of distant metastasis than 2 out of 39 patients (5.1%) in the low-lncATB-expression group (*χ*^2^ = 4.413, P = 0.036). Although there were no significant differences in the clinical stage (*P* = 0.060) or the number of lymph node (LN) metastases (*P* = 0.051) between the low- and high-lncATB-expression groups, we believe that there would be different results if we expanded the data. However, no significant differences in the years of age, tumour size or lymph node status were identified between the low-lncATB-expression group and the high-lncATB-expression group.

**Table 1:** Clinicopathological characteristics of the breast cancer patients according to lncATB expression

Characteristics	lncATB expression		*χ* ^2^	*P* value
	High *n* (%)	Low *n* (%)		
Age years				
≤40	14 (15.2)	4 (10.3)	0.569	0.451
>40	78 (84.8)	35 (89.7)		
Histology grade				
I and II	30 (32.6)	22 (56.4)	6.482	**0.011**
III	62 (67.4)	17 (43.6)		
Stage				
I and II	55 (59.8)	30 (76.9)	3.532	0.060
III and IV	37 (40.2)	9 (23.1)		
Tumour size				
T0-T1	20 (21.7)	9 (23.1)	0.028	0.866
T2-T4	72 (78.3)	30 (76.9)		
LN status				
Negative	38 (41.3)	19 (48.7)	0.612	0.434
Positive	54 (58.7)	20 (51.3)		
No. of LN metastasis				
LN ≤ 3	63 (68.5)	31 (79.5)	5.965	0.051
3 <LN <10	12 (13.0)	7 (17.9)		
LN ≥ 10	17 (18.5)	1 (2.6)		
Distant metastasis				
Negative	74 (80.4)	37 (94.9)	4.413	**0.036**
Positive	18 (19.6)	2 (5.1)		
Ki67				
Negative	7 (7.6)	17 (43.6)	23.696	**<0.001**
Positive	85 (92.4)	22 (56.4)		

These bold values indicate there is statistical significance between the two groups, *P* < 0.05 or *P* < 0.01.

Since we found that the sponging of miR-200c by lncATB restored the expression of Twist1 in breast cancer cell lines, we also investigated the correlation between the clinicopathological characteristics and Twist1 expression levels in breast cancer patients. As shown in Supplementary Tables S2, more breast cancer patients were stage III or IV (39 out of 86, 45.3%) when Twist1 displayed a high expression level, while fewer breast cancer patients were at these stages (7 out of 45, 15.6%) when Twist1 displayed a low expression level (*χ*^2^ = 10.239, *P* = 0.001). Moreover, breast cancer patients were more likely to have lymph node metastasis when Twist1 was highly expressed (*χ*^2^ = 7.523, *P* = 0.023). Nevertheless, no significant differences in age, histology grade, tumour size, distant metastasis or Ki67 were identified between the low- and high-Twist1-expression groups.

## Discussion

**Fig. 8:**
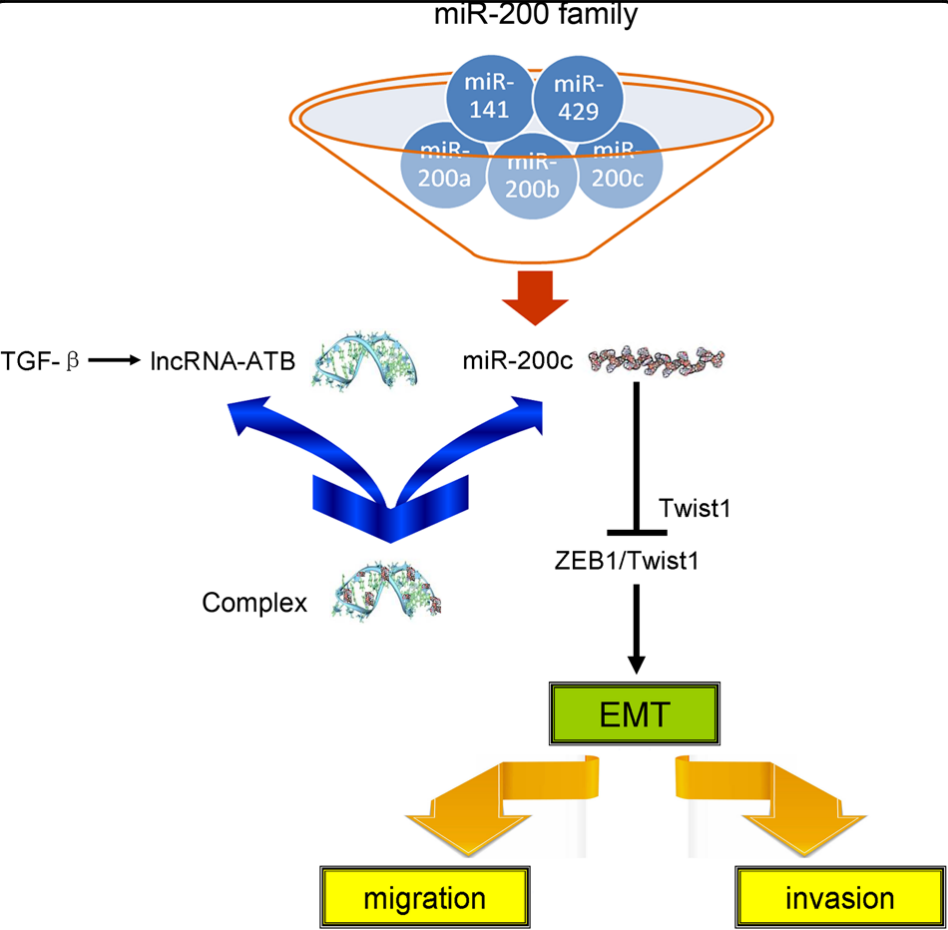
A schematic model of how lncATB promotes the invasion-metastasis cascade in breast cancer. LncATB, which is activated by TGF-β, upregulates Twist1 by competitively binding miR-200 family members, especially miR-200c, and then induces the EMT, which promotes breast cancer cell migration and invasion

In the present study, we investigated the role of lncATB, an oncogenic lncRNA, in breast cancer cells and breast cancer patients, especially, its critical regulatory mechanism in breast cancer cell migration and cancer metastasis. We found that lncATB was induced by the TGF-β treatment of breast cancer cells, whose induction is associated with EMT transition. Specifically, our study showed that lncATB could directly bind to the miR-200 family, with an especially high binding affinity for miR-200c. Moreover, lncATB regulated the gene expression of transcription factor Twist1, by acting as a competitive sponge for miR-200c and further promoted breast cancer cell migration and invasion (Fig. 8). Additionally, lncATB also increased breast cancer cell proliferation and promoted breast cancer cell clonality.

To date, several lncRNAs have been found to regulate breast cancer initiation and progression, and some of these lncRNAs including lncATB are potential prognostic markers for breast cancer^25^. Recent studies have indicated that lncATB is an essential modulator of cancer cell proliferation^36^, the EMT^32,36,43^, anti-apoptosis^33^, migration and invasion^32,34–36,42,43^. A previous report demonstrated that lncATB promoted EMT-mediated trastuzumab resistance^45^. Consistent with that report, we also confirmed that lncATB promoted the EMT by inducing the expression of mesenchymal hallmarks, such as N-Cad and Vimentin, and inhibiting the expression of the epithelial hallmark E-Cad in breast cancer cell lines. The ectopic expression of lncATB in breast cancer cells led to increased cellular proliferation, colony formation, migration and invasion, while lncATB knockdown inhibited those functions.

Emerging evidence has indicated that lncATB can promote cancer cell invasion and metastasis in a variety of cancer cells by acting as a competitive endogenous RNA (ceRNA) that acts as a sponge and modulates the functions of the miR-200 family by enhancing the expression of miR-200 target genes, such as *ZEB1, ZEB2 or TGF-β2*^32,34,42,43^. In our study, we showed that, by sponging miR-200c, lncATB facilitated the expression of Twist1, resulting in promoted tumour progression. With regards to the miR-200 family members, miR-200c and then miR-200b was highly inhibited with overexpression of lncATB, but rather than miR-200a as described in a previous report in HCC^32^. Several studies reveal that the member of miR-200 family showing the strongest binding ability with lncATB was different depending on tumour types such as miR-200a in liver cancer^32^, miR-200b in oesophageal squamous cell carcinoma^41^ and miR-141 in gastric cancer^39^. Together with our finding on the binding between miR-200c and lncATB in breast cancer, these findings suggest that lncATB might modulate different spectrum of miR-200s family. Probably in breast cancer, lncATB does mainly bind to miR-200c for silencing its effects on target genes.

Intriguingly, *Twist1* was another most significantly activated transcriptional factors by lncATB overexpression among those evaluated in breast cancers. Twist1 was induced at both mRNA and protein levels by lncATB, while both of them were downregulated by depletion of lncATB. In contrast to the previous study that both ZEB1 and ZEB2 were activated in HCC, we found that Twist1 is another transcription factor induced by lncATB not reported previously. The positive association between lncATB and Twist1 was further confirmed in resected tissues from tumour xenografts. Notably, we also found that Twist1 expression correlated with lncATB in the tissues from breast cancer patients, keeping consistent with their correlation found in breast cancer cells. Taken together, these results suggested that lncATB promotes the EMT process by upregulating Twsit1. Remarkably, the results of mouse mammary-pad injection experiments with stable shATB-expressing MDA-MB-231 cells indicated the abolishment of breast cancer growth in vivo. All of these studies implied that lncATB might play a crucial role in breast cancer tumourigenesis and progression.

Several previous reports have shown that lncATB is upregulated in patients with cancer, especially metastatic cancer, including renal cell carcinoma^36,37^, hepatocellular carcinoma^32^, non-small cell lung cancer^33^, osteosarcoma^34^, colon cancer^35^, gastric cancer^38,39^, oesophageal squamous cell carcinoma^41^, glioma malignancy^42^ and prostate carcinoma^43^. In the current study, lncATB and its downstream gene *Twist1* have been applied to the association with the clinicopathological characteristics and survival of breast cancer patients. Notably, for the first time, we demonstrated that lncATB was highly expressed in clinical breast cancer tissues, especially in luminal B, Her-2 and TNBC subtypes compared to the luminal A subtype, which keeps consistent with that found in breast cancer cells. Furthermore, high levels of lncATB in breast cancer patients were associated with more distant metastasis, higher histological grade, more lymph nodes involvement, and higher Ki67 levels, i.e., high aggressiveness of breast cancers. In addition, the patients with high lncATB level showed a poor clinical outcome including both DFS and OS, similar to those found in carcinomas^34,37,38,42,43,46,47^. Taken together, the findings here demonstrated that lncATB associates with unfavourable biological behaviour and predicts poor prognosis in breast cancers. Additionally, our study also showed that the AUC of the ROC analysis of lncATB was as high as 0.851 in breast cancer patients, indicating the diagnostic value of lncATB for cancer. Collectively, the ability of lncATB to predict poor clinical outcomes is promising, and the results suggest that lncATB may be used as a novel marker in cancer diagnosis and predicting prognosis.

In conclusion, we have described the important regulatory roles played by the lncATB/miR200c/Twist1 axe in breast cancer carcinogenesis and progression. Our study suggests that addressing the uncharted potential effects of lncATB will have a fundamental influence on breast cancer biology, especially in the search for novel diagnostic markers for breast cancer as well as new therapeutic targets and pharmaceutical interventions.

## Materials and methods

### Patients

A total of 131 primary breast cancer tissue samples and 16 normal breast tissue samples were collected from patients who underwent surgeries in the Breast Center at the Cancer Hospital of Shantou University Medical College between September 2010 and June 2016. Tissue specimens obtained during surgery were immediately snap-frozen in liquid nitrogen, and RNA was extracted within 48 h. None of the patients had received preoperative chemotherapy, radiotherapy, endocrine therapy, or targeted therapy. Clinicopathological parameters, such as the tumour size, lymph node status, and pathological features, were recorded from the patients’ medical records. The study was approved by the ethics committee of the Cancer Hospital of Shantou University Medical College and written informed consent was obtained from all patients involved.

### Cell lines and cell culture

The MCF-7, T47D, MDA-MB-231, BT-549, BT-20 MDA-MB-436, MDA-MB-435, and SKBR3 breast cancer cell lines and MCF-10A normal breast cells were purchased from the American Type Culture Collection (ATCC, USA), which then were authenticated by STR (Short Tandem Repeat) profiling and tested for mycoplasma-free contamination. All the cells were maintained in dulbecco's modified eagle medium (DMEM) containing 10% foetal bovine serum (FBS) (Gibco/Life Technologies, Carlsbad, CA, USA) and were cultured in a humidified 5% CO_2_ incubator at 37 °C. The medium was replaced every 2 days.

### RNA extraction and real-time qPCR

Total RNA from all the tissues and cells was extracted with the TRIzol reagent (Life Technologies, Gaithersburg, MD, USA). The RNA was reverse-transcribed into cDNA using the Prime-Script RT-PCR kit (Takara, Dalian, China) following the manufacturer’s instructions. All the primer sequences are shown in Supplementary Table S3. The transcript levels of lncATB and the EMT markers were analysed using real-time PCR assays (SYBR Premix Ex Taq™, TaKaRa, Dalian, China) in triplicate by reacting the cDNA with sequence-specific PCR primers in an ABI PRISM 7500 sequence detection PCR system (Applied Biosystems, Foster City, CA, USA). The relative expression levels of lncATB and the mRNAs were calculated and normalized relative to β-actin using the 2-ΔΔCt method.

### RNA pull-down assay

RNA pull-down assays were performed according to the manufacturer’s instructions (Sagene Technology Inc., Guangzhou, China). We labelled lncATB probes with biotin using transcription and then incubated the probes with an MCF-7 cytoplasmic lysate to form lncRNA-microRNA complexes. The complexes were combined via chain affinity with magnetic beads and thus separated from other components. After complex elution, we determined by qRT-PCR assays the miR-200 family members that were pulled down.

### Western blot analysis

The total cellular proteins were lysed in RIPA buffer (Cell Signaling Technology, Inc., MA, USA) supplemented with protease inhibitors. The protein concentration was determined using a BCA Protein Assay Kit (Beyotime Biotechnology, Shanghai, China). Extracted protein (30–50 μg) was separated by 10% SDS polyacrylamide gel electrophoresis and transferred to a polyvinylidene difluoride membrane (EMD Millipore Corporation, Billerica, MA, USA). The membrane was blocked with 5% non-fat milk in Tris-buffered saline with 0.1% Tween-20 (TBST) for 1 h and then probed with the indicated primary antibodies (Supplementary Table S4) at 4 °C overnight with gentle shaking. The following day, the membrane was washed with TBST (5 min × 3) and then incubated in secondary antibody (Supplementary Table S5) for 2 h at room temperature. Then the immunocomplexes were detected using the ECL Plus reagent (Applygen Technologies, Inc., Beijing, China).

### Immunofluorescence assay

MCF-7 or BT-549 cells were plated on glass slides (1 × 10^4^ cells/well) and incubated for approximately 24 h at 37 °C. After treating the cells with formaldehyde and Triton-X, the cells were incubated with primary antibodies directed against E-Cad and Vimentin (dilution 1:250, Cell Signaling Technology, Inc., USA) at 4 °C overnight with gentle shaking. The following day, the cells were incubated with secondary antibody (Supplementary Table S5) for 1 h at room temperature away from light sources. Finally, the samples were stained with 4',6-diamidino-2-phenylindole (DAPI) and were observed with a TS100/100-F inverted fluorescence microscope (Nikon, Corporation, Tokyo, Japan).

### Cell proliferation assay

Cells were seeded into 96-well plates at a density of 1×10^3^ cells/well and incubated for 1−5 days. Every 24 h, 10 μl of Cell Counting Kit-8 solution (CCK-8, Dojindo, Japan) was added to each well, and the plates were incubated at 37 °C in a humidified atmosphere containing 5% CO_2_ for 2 h. Then, the optical density (OD) of each sample was determined by measuring the absorbance at 490 nm with a microplate reader (BioTek Instruments, Inc., Winooski, VT, USA).

### Colony formation assay

Approximately 1×10^3^ viable cells were seeded into 100-mm plates and incubated in DMEM with 10% FBS at 37 °C. After 14 days, when the colonies were larger than 50 cells, the cells were stained using crystal violet. Each experiment was repeated twice.

### Wound-healing assay

Cells (5×10^5^) were seeded into six-well plates and allowed to grow to 90–95% confluence. Subsequently, the cells were scraped with a 200-μl plastic tip to generate straight wounds. At 0 and 48 h after wounding, images were recorded with an inverted research eclipse TS100/100-F microscope (Nikon, Corp., Tokyo, Japan).

### Cell migration and invasion assay

Cell migration was determined by using 24-well Transwell Chambers with an 8-μm pore size and a track-etched membrane (Corning, Inc., New York, CA, USA). A chamber insert was placed into each well of a 24-well dish containing 600 μl of DMEM supplemented with 20% FBS in the lower chamber. Approximately 5×10^4^ cells were suspended in 200 μl of serum-free DMEM and seeded into the upper chamber. The cells were incubated at 37 °C with 5% CO_2_ for 24 h. Then, the cells on the lower chamber membrane were fixed with 100% methanol for 30 min at room temperature and stained with 0.1% crystal violet for 20 min at room temperature. Subsequently, the nonmigrated cells on the upper side of the membranes were removed, and the migrated cells on the underside of the membranes were observed with a TS100/100-F inverted fluorescence microscope (Nikon, Corporation, Tokyo, Japan) using five randomized fields. A cell invasion assay was also conducted in the same manner but with Matrigel™ Invasion Chamber 24-well plates with 8.0-μm pores (BD Biosciences, San Jose, USA) and an incubation time of 48 h.

### RNA interference

The miR-200 family member mimics and inhibitor were synthesized by GenePharma (Shanghai GenePharma Co., Ltd., Shanghai, China). The siRNA sequences for the miR-200 family members are listed in Supplementary Table S6. The day prior to transfection, 5×10^5^ MCF-7 or BT-549 cells were inoculated into each well of a six-well culture plate. For the transfection, 75 pmol of siRNA was combined with 5 μl of Lipofectamine 3000 (Invitrogen; Thermo Fisher Scientific, Inc., Waltham, MA, USA) according to the manufacturer’s protocol.

### Tumour xenografts

All animal studies were approved by the Institutional Animal Care and Use Committee of Shantou University Medical College and were performed with 4-week-old female nude mice (Vital River Laboratory Animal Technology Co., Ltd., Beijing, China). A total of 2×10^6^ cells were suspended in 100 μl of phosphate buffer saline (PBS) and injected into the fourth breast of three female nude mice in each group. Tumour growth was determined, along the longest diameter and shortest width, every 3 days using digital callipers. The tumour volume was calculated according to the following formula: Volume = 0.5 × width^2^ × length. Finally, the tumours were removed, measured and weighed. For xenograft animal studies, the protocol was approved by the Animal Care and Use Committee of Shantou University Medical College.

### Vector and shRNA construction

The cDNA encoding lncATB was amplified via PCR with the TransStart FastPfu DNA Polymerase (TransGen Biotech, Beijing, China, #J30702). Then, the lncATB sequence was subcloned into the *Xho*I and *Eco*RI sites of the pcDNA3.1 (+) vector, which was named pcDNA3.1-ATB (ATB) (Supplementary Fig. S1a). The lncATB primers were 5′-CCCTCGAGCCCTGGGGCTCTGCAATTG-3′ (forward) and 5′-CGGAATTCGGTAAATGAGTCCAAAG TCATACTGCCC-3′ (reverse).

The design of shATB was assisted by GenePharma (Shanghai GenePharma Co., Ltd., Shanghai, China) and included the following primers: shATB-#1, 5′-CCTTATGGCCTAGATTACCTTTCCAT-3′; shATB-#2, 5′-CCTGTCTGTATTT GCGAATACCTT-3′; and shNC, 5′-T TCTCCGAACGTGTCACGT-3′. The shATB sequences were cloned into pGPU6/GFP/Neo plasmids, which are driven by a U6 promoter.

### Luciferase reporter assays

The lncATB fragment containing the predicted miR-200 family member binding site was obtained, and the putative binding site sequences were cloned into a pcDNA3.1(+)/Luc2 = tdT vector (Addgene, Sidney St., Cambridge, MA) (Supplementary Fig. S6a). The twist1–3′ UTR fragment sequence containing the binding site with miR-200c was cloned into the pMIR-reporter vector (Addgene, Sidney St., Cambridge, MA) (Supplementary Fig. S6b). Cells were cotransfected with luciferase reporters and other plasmids or short RNAs (ATB, shATB, miR-200 family mimics, or the miR-200 family inhibitor) using Lipofectamine 3000 (Invitrogen; Thermo Fisher Scientific, Inc., Waltham, MA, USA). After 48 h, a luciferase assay was performed using the Dual-Luciferase Reporter Assay System (Promega, Madison, WI, USA) according to the manufacturer’s protocol.

### Statistical analysis

Statistical analysis was performed using the SPSS version 20.0 (SPSS, Inc., Chicago, IL, USA) and Prism V5.0 (GraphPad Software, Inc., La Jolla, CA, USA) software. The experimental data were presented as the means ± standard deviation (SD). Statistical analysis was performed using Student’s *t* test. The Spearman correlation coefficient was used to assess correlations between lncATB expression and clinical features. The survival analysis was performed using the log-rank test in GraphPad Prism 5. A value of *P* < 0.05 was considered to indicate a statistically significant difference. The corresponding significance levels are indicated in the figures.

## Electronic supplementary material

Supplementary information
