# Supplementary material for: Long noncoding RNA ATB promotes the epithelial−mesenchymal transition by upregulating the miR-200c/Twist1 axe and predicts poor prognosis in breast cancer
Source: Cell Death Dis. 2018 Dec 5;9(12):1171. doi: 10.1038/s41419-018-1210-9 (PMC6281614; doi:10.1038/s41419-018-1210-9)
Supplement: Supplementary file 1 — Supplementary information [file 41419_2018_1210_MOESM1_ESM.docx]

**Supplementary Information**

Supplementary Information includes six figures and six tables as followed:


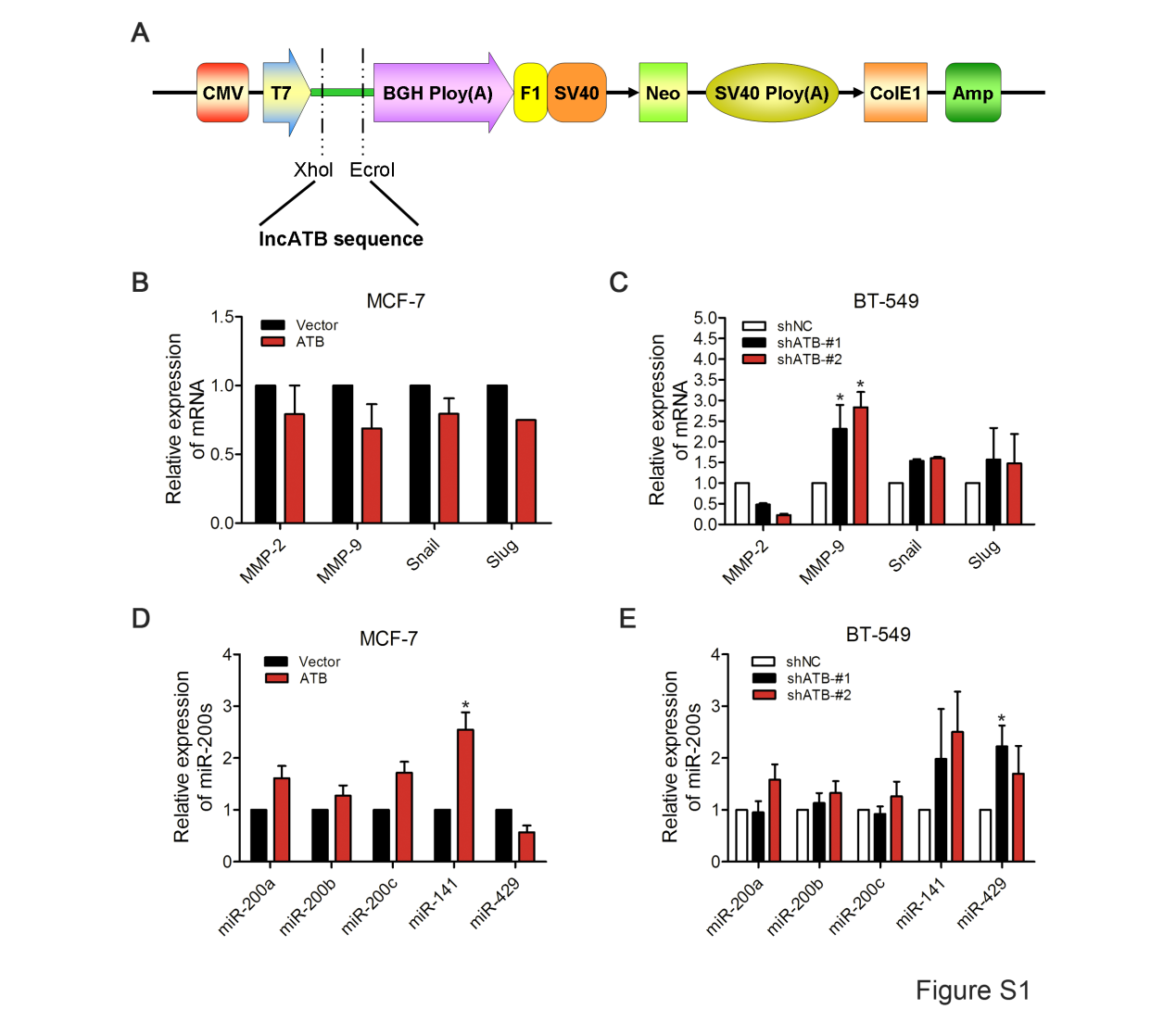


**Supplementary Fig. S1:** Construction of lncATB plasmid, relative expression of EMT markers and miR-200s in MCF-7-ATB or BT-549-shATB cells. **a** The sequence of lncATB (ENST00000493038) was inserted into the vector pcDNA3.1(+). **b** Relative expression of EMT markers in MCF-7-ATB and control MCF-7 cells. **c** Relative expression of EMT markers in BT-549-shATB and control BT-549-shNC cells. **d** Relative expression of miR-200s in MCF-7-ATB and control MCF-7 cells. **e** Relative expression of miR-200s in BT-549-shATB and control BT-549-shNC cells. *P<0.05.


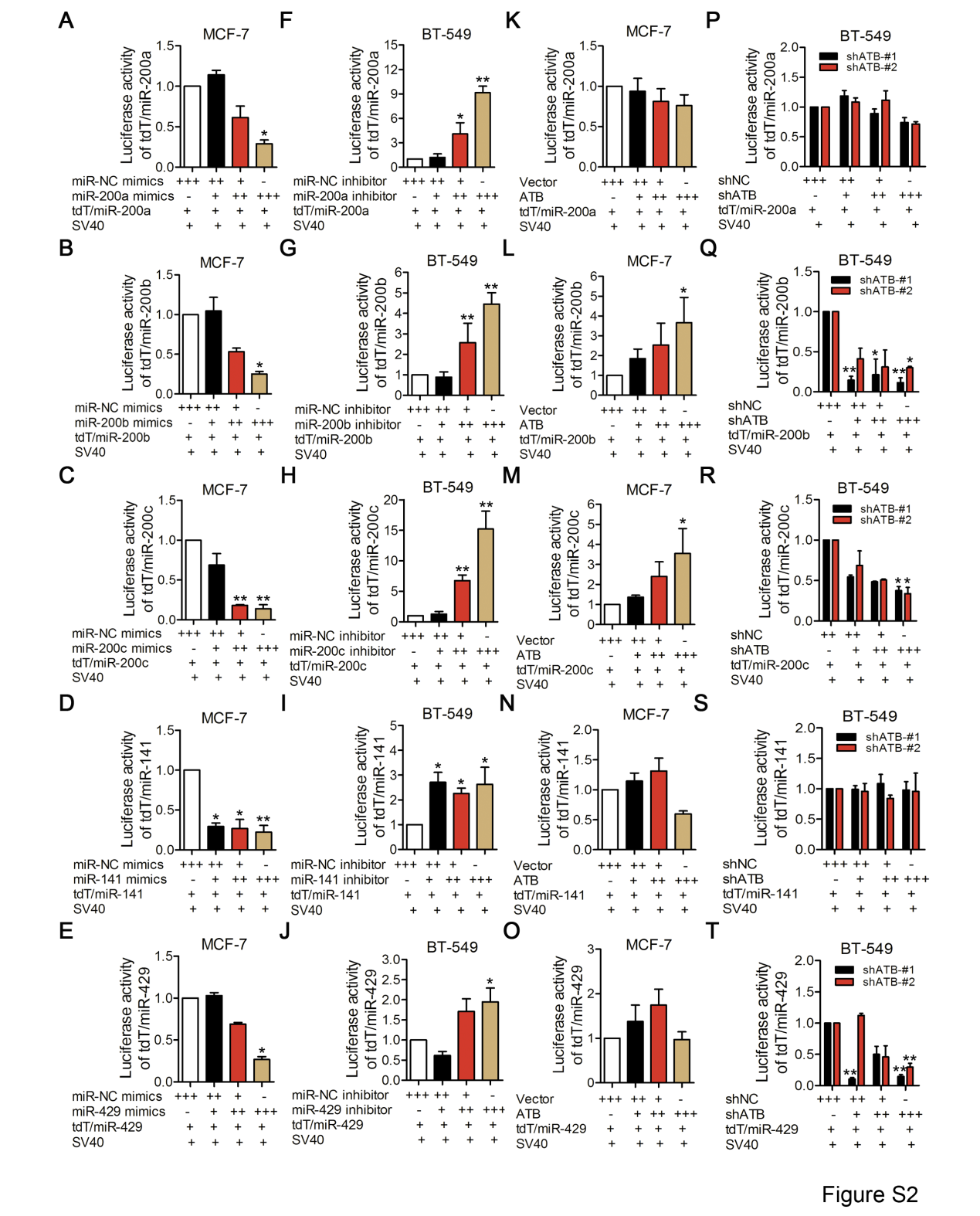


**Supplementary Fig. S2:** The tdT/miR-200s luciferase reporters are activity with a dose dependent manner. **a-e** Luciferase activity in MCF-7 cells cotransfected with miR-200s mimics and luciferase reporters, containing (a) miR-200a, (b) miR-200b, (c) miR-200c, (d) miR-141, and (e) miR-429, with a dose dependent. **F-j** Luciferase activity in BT-549 cells cotransfected with miR-200s inhibitor and luciferase reporters, containing (f) miR-200a, (g) miR-200b, (h) miR-200c, (i) miR-141, and (j) miR-429, with a dose dependent. **k-o** Luciferase activity in MCF-7 cells cotransfected with pcDNA3.1-ATB and luciferase reporters, containing (k) miR-200a, (l) miR-200b, (m) miR-200c, (n) miR-141, and (o) miR-429, with a dose dependent. **p-t** Luciferase activity in BT-549 cells cotransfected with shATB-#1/2 and luciferase reporters, containing (p) miR-200a, (q) miR-200b, (r) miR-200c, (s) miR-141, and (t) miR-429, with a dose dependent. *P<0.05, **P<0.01.


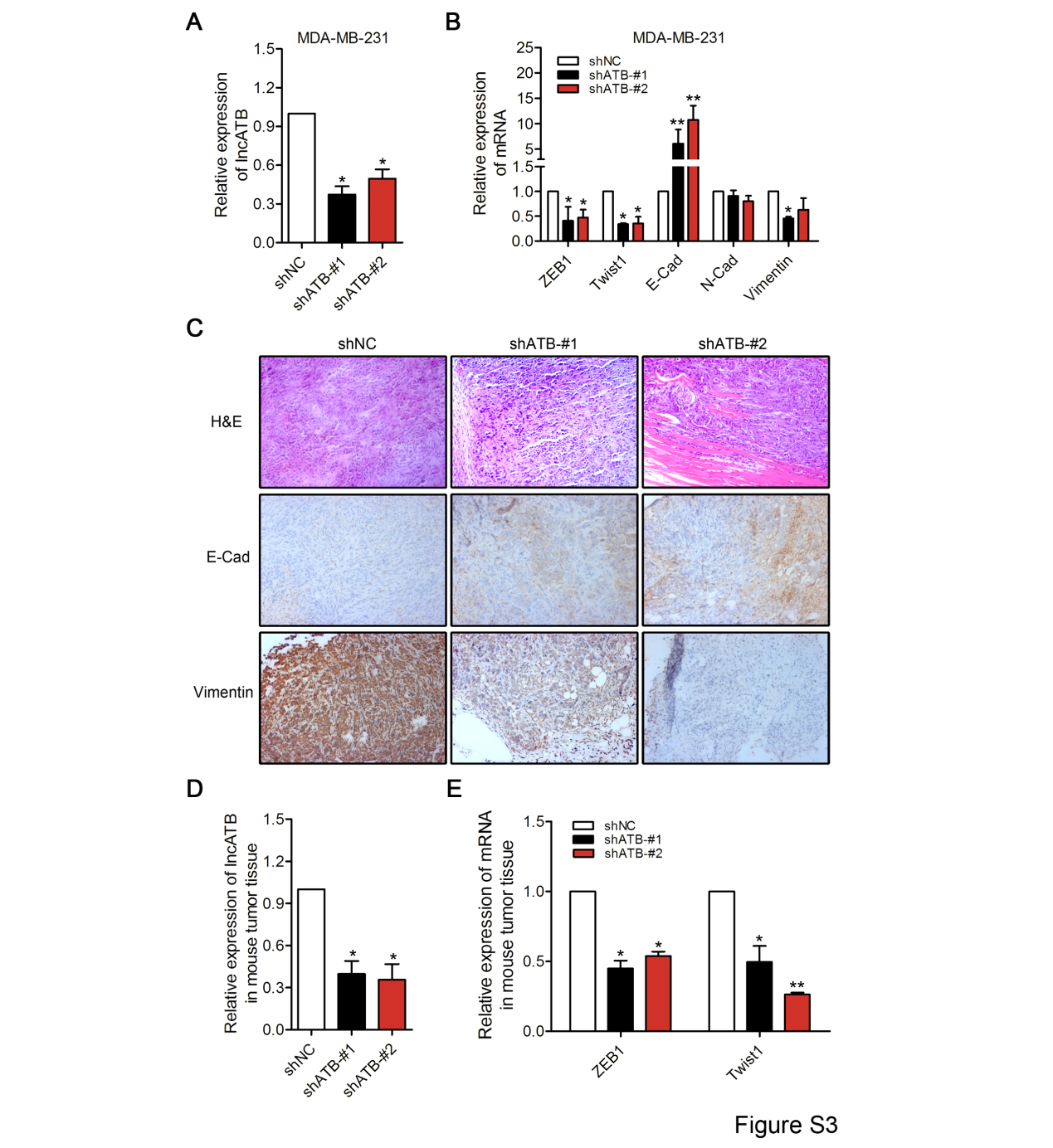


**Supplementary Fig. S3:** Relative expression of lncATB and EMT markers in MDA-MB-231 cell and mouse tumor tissues. **a** Knocking down lncATB in stably MDA-MB-231 cell clones. **b** The mRNA levels of EMT markers in MDA‑MB‑231 cells which were knocked down lncATB. **c** Hematoxylin-eosin and immunohistochemical staining of E-Ca and Vimentin in mouse tumor tissues. **d** The lncATB levels in mouse tumor tissues with control and low-expression lncATB. **e** The relative expression of EMT markers in mouse tumor tissues with control and low-expression lncATB.

**
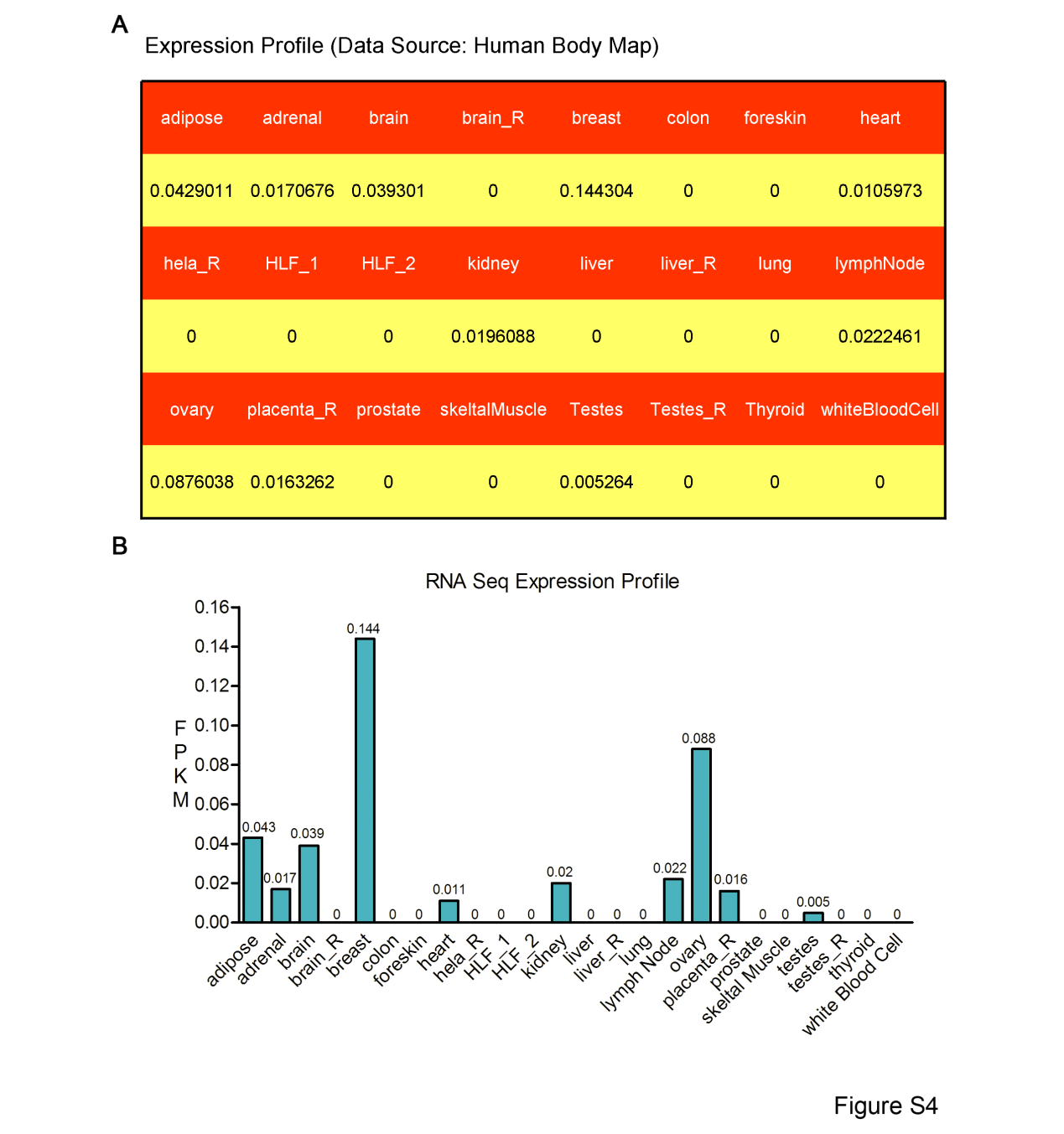
**

**Supplementary Fig. S4:** Expression profile of lncATB in human body map on database NONCODE. NONCODE TRANSCRIPT ID: NONHSAT168138.1, NONCODE Gene ID: NONHSAG068618.

**
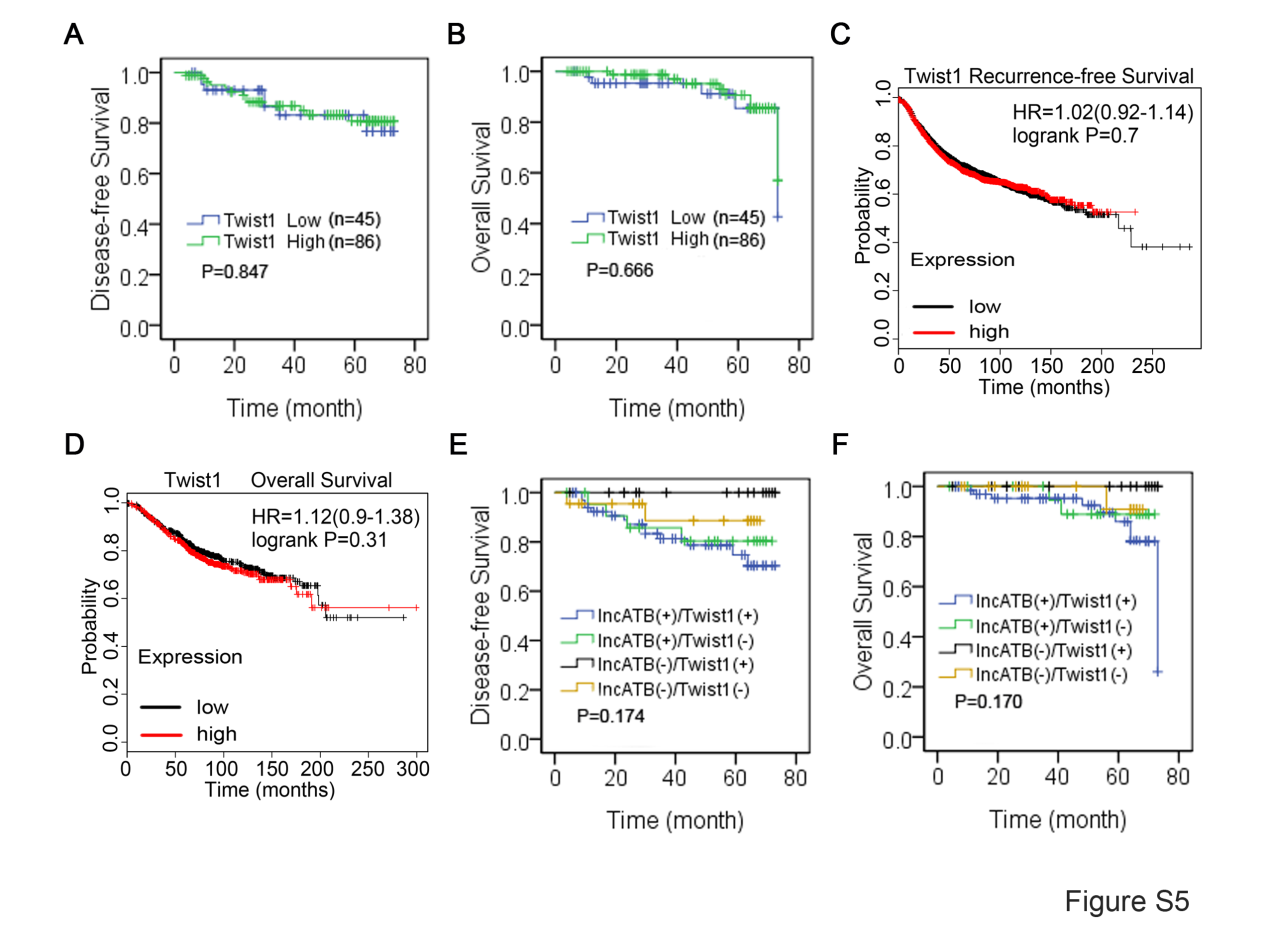
**

**Supplementary Fig S5:** The prognosis of Twist1 and lncATB in breast cancer. **a** Disease-free survival for two groups defined by low and high expression of Twist1 in breast cancer patients. **b** Overall survival for two groups defined by low and high expression of Twist1 in breast cancer patients. **c** Recurrence-free survival (RFS) in breast cancer patients according to Twist1 expression in Kaplan Meier Plotter database. **d** Overall survival (OS) in breast cancer patients according to Twist1 expression in Kaplan Meier Plotter database. **e** Disease-free survival for four groups defined by low and high expression of Twist1 and lncATB in breast cancer patients. **f** Overall survival for four groups defined by low and high expression of Twist1 and lncATB in breast cancer patients.


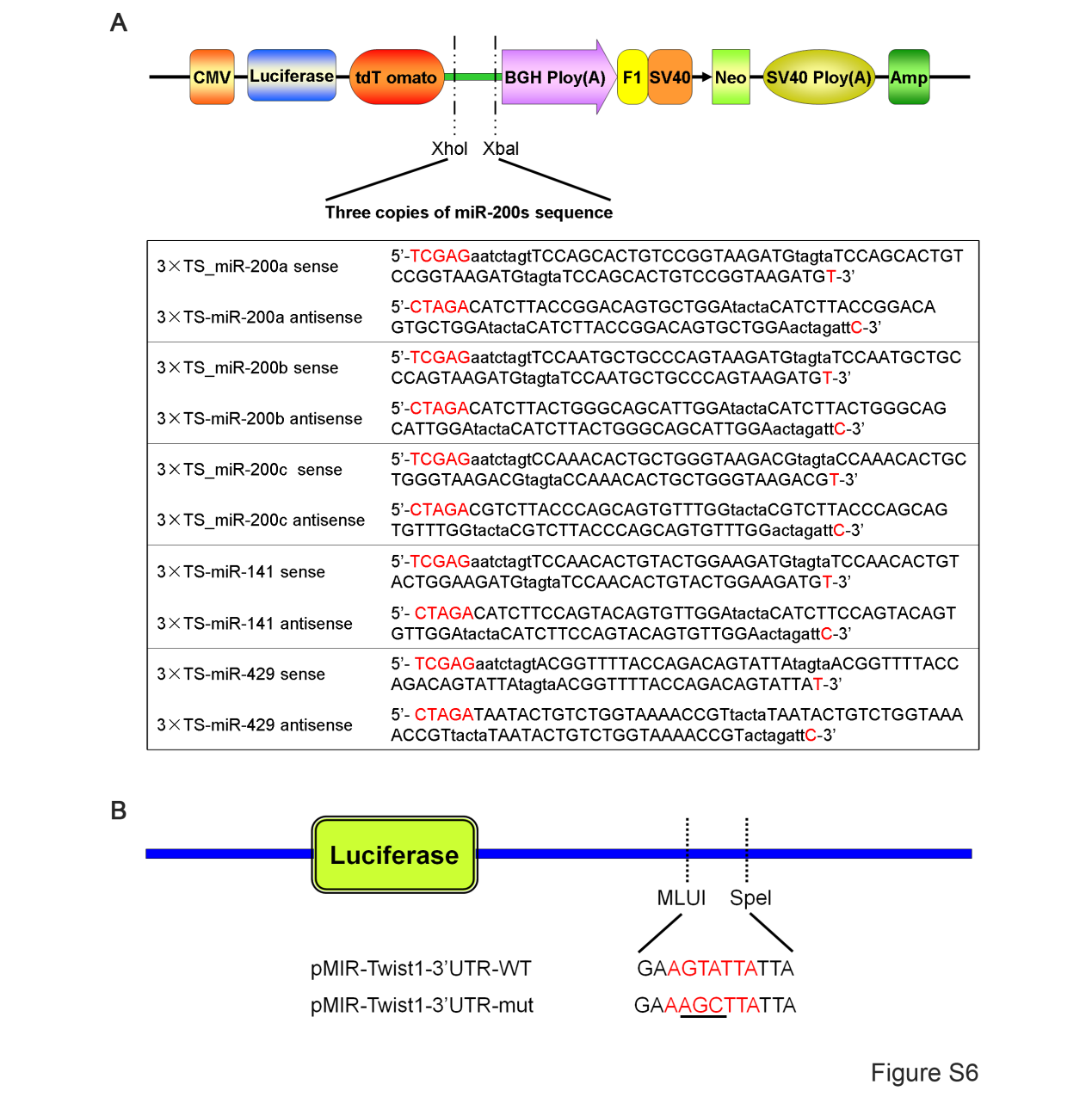


**Supplementary Fig. S6:** Construction of the luciferase reporters. **a** Three copies of miR-200s sequence was inserted into the vector pcDNA3.1(+)/Luc2=tdT. **b** The twist1-3’UTR-WT or twist1-3’UTR-mut sequence was inserted into the vector pMIR-reporter.

**Supplementary Table S1. Schematic outlining the predicted binding sites of**

**miR-200c on ZEB1 and Twist1**


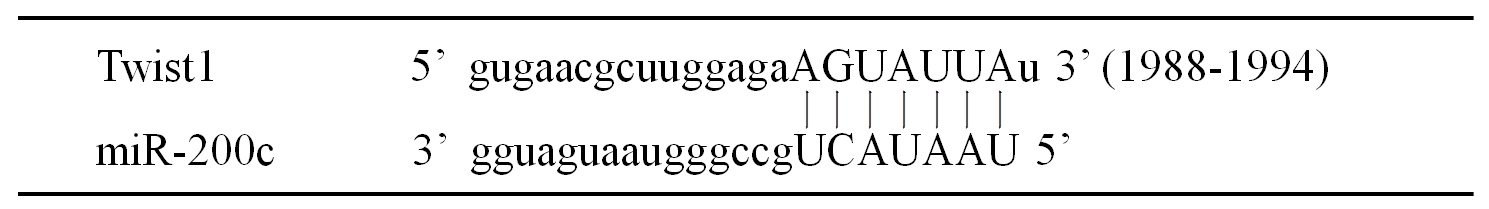


**Supplementary Table S2. Clinicopathological characteristics of the breast cancer patients according to Twist1 expression**


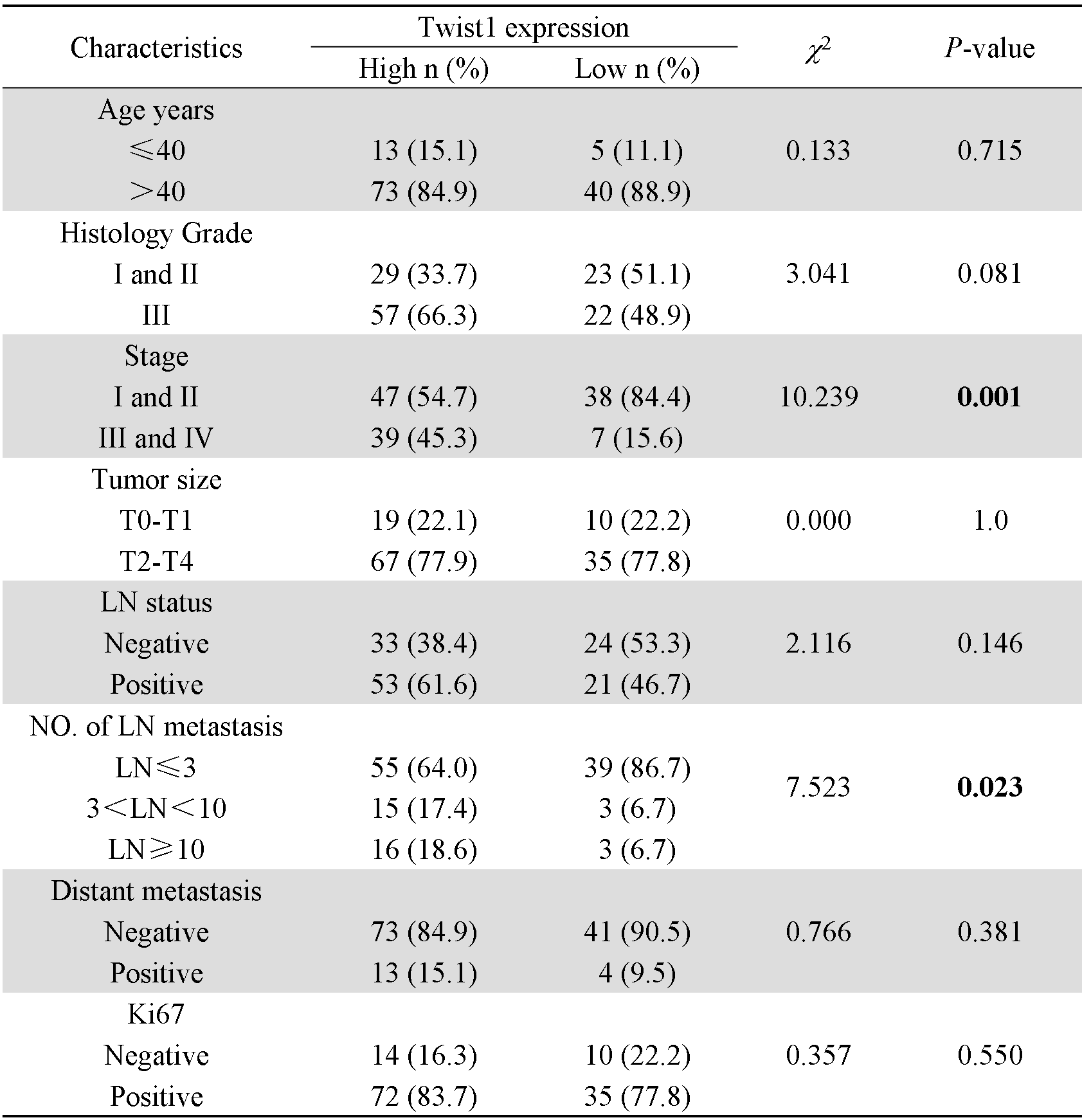


**Supplementary Table S3. Primers used for reverse transcription and qRT-PCR**

**
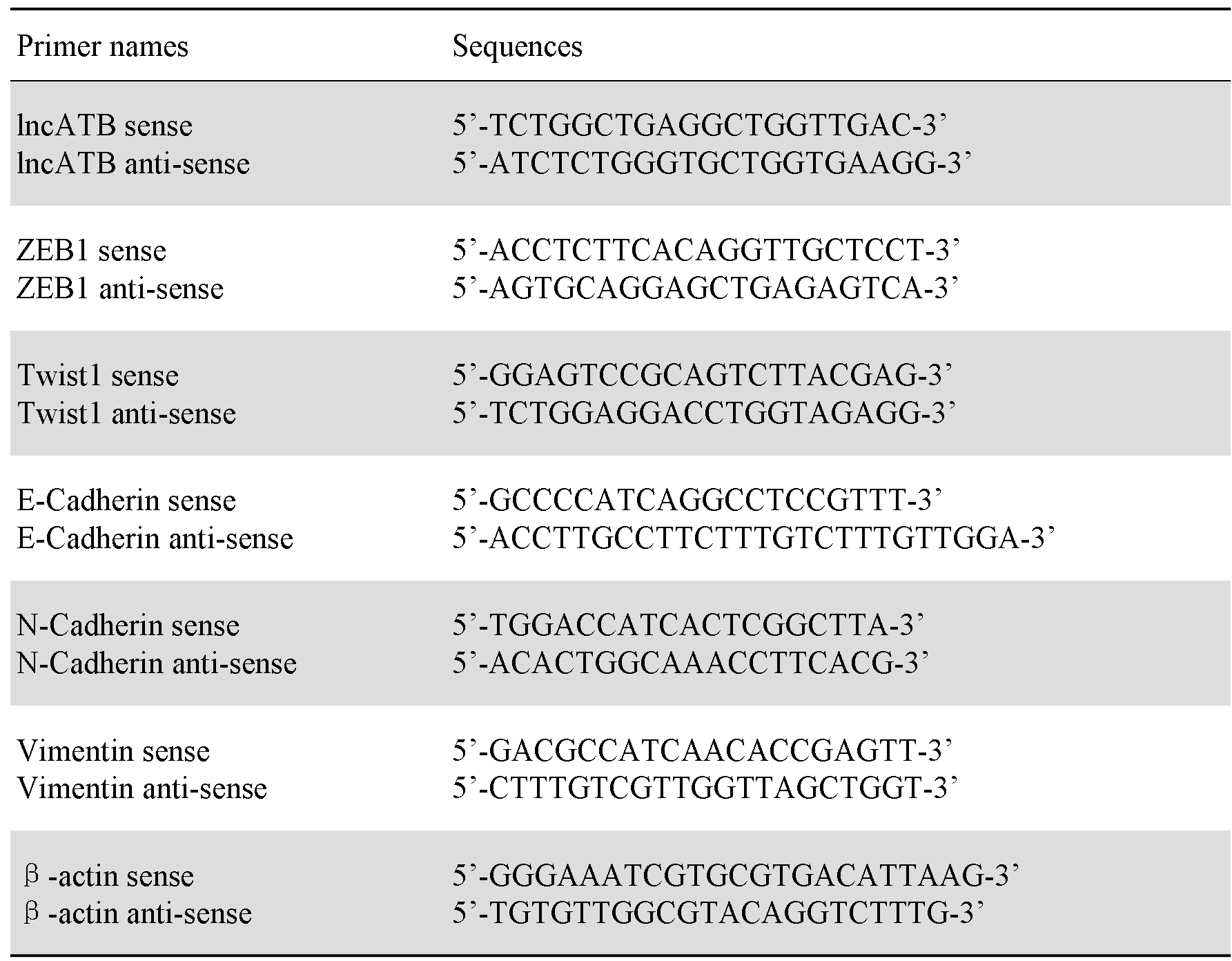
**

**Supplementary Table S4. Primary antibodies for Western blot**

**
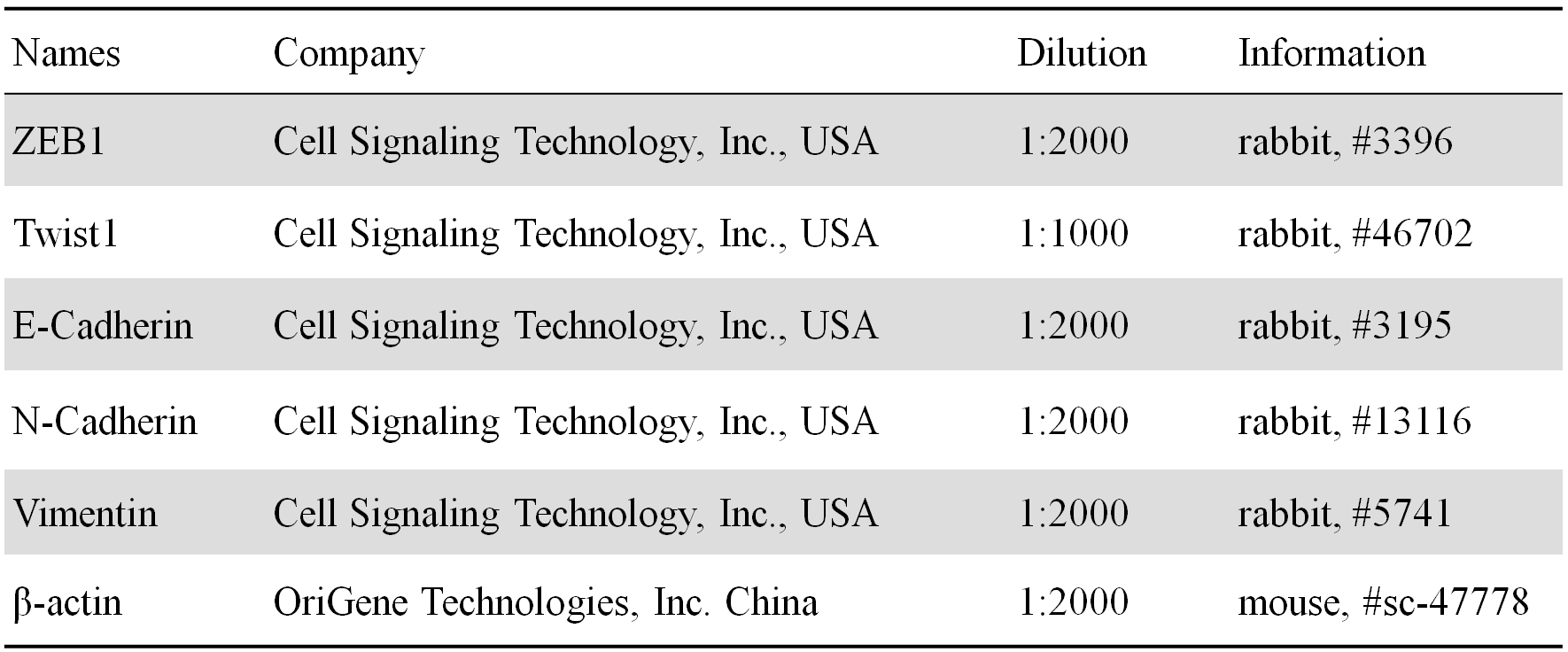
**

**Supplementary Table S5. Secondary antibodies for Western blot**

**
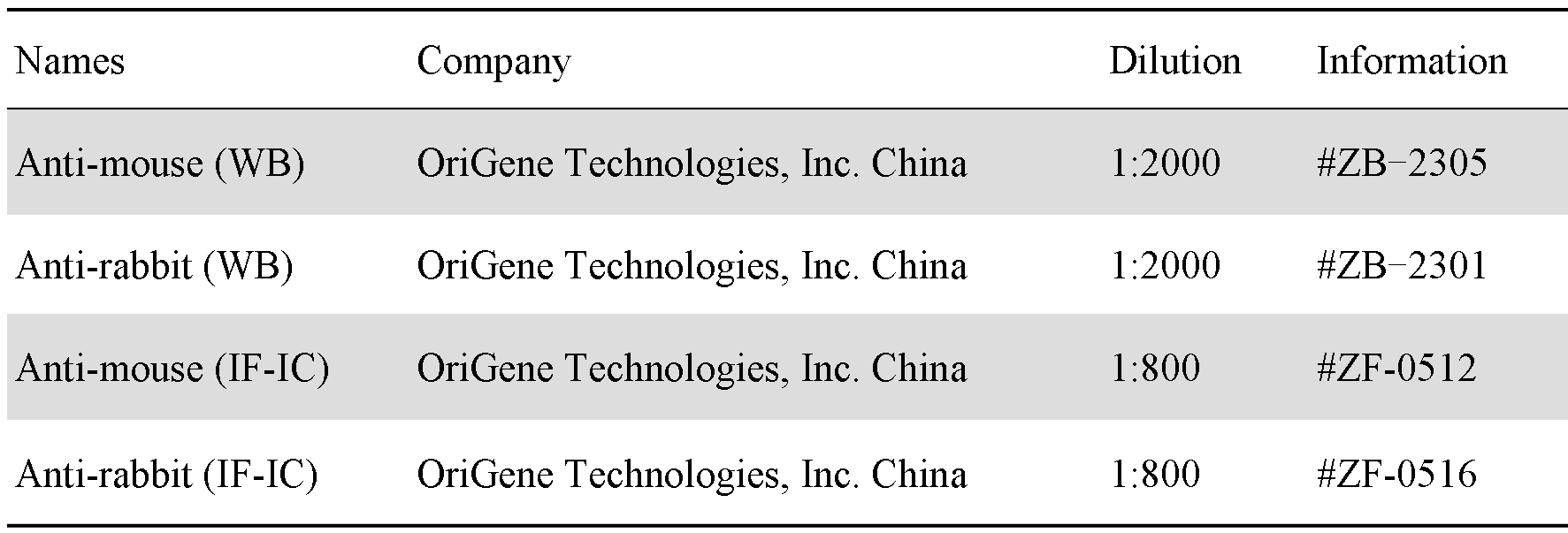
**

**Supplementary Table S6. siRNA sequences**

**
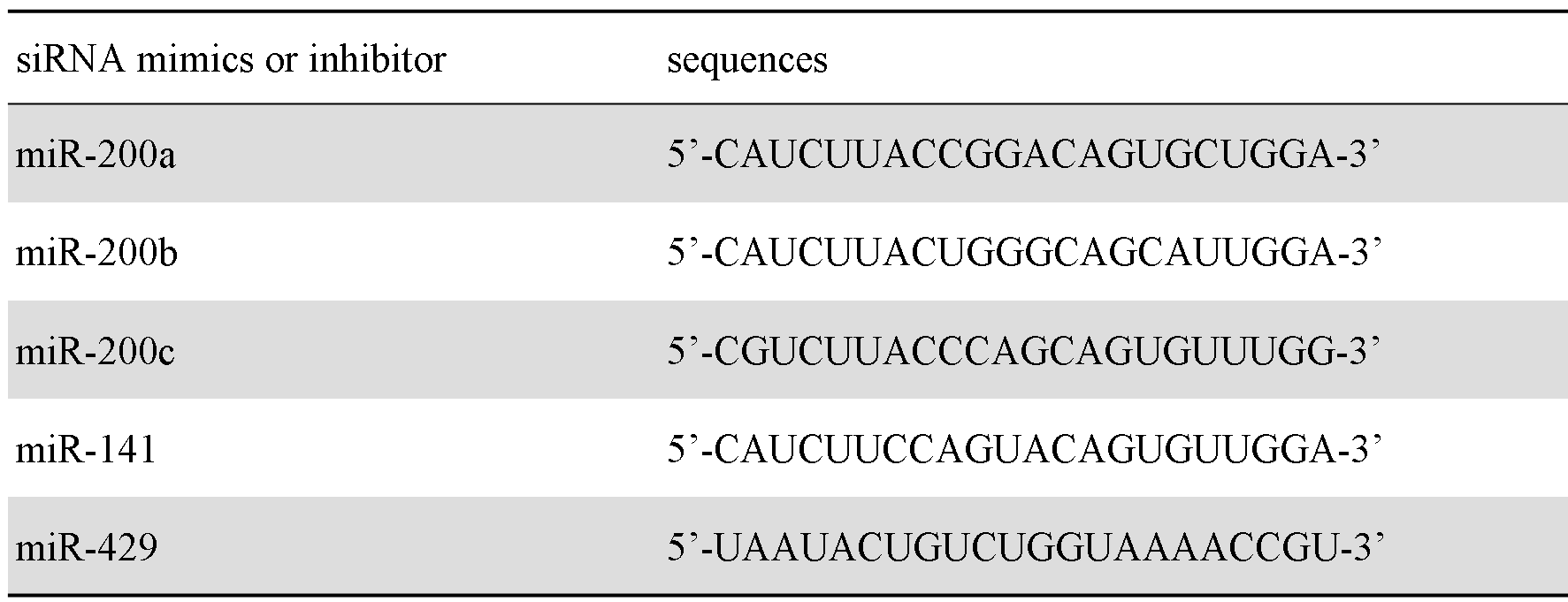
**
